# Functional Annotation of the *Ophiostoma novo-ulmi* Genome: Insights into the Phytopathogenicity of the Fungal Agent of Dutch Elm Disease

**DOI:** 10.1093/gbe/evu281

**Published:** 2014-12-24

**Authors:** André M. Comeau, Josée Dufour, Guillaume F. Bouvet, Volker Jacobi, Martha Nigg, Bernard Henrissat, Jérôme Laroche, Roger C. Levesque, Louis Bernier

**Affiliations:** ^1^Institut de Biologie Intégrative et des Systèmes (IBIS), Université Laval, Québec, Québec, Canada; ^2^Centre d’Étude de la Forêt (CEF), Université Laval, Québec, Québec, Canada; ^3^Institut de Recherches Cliniques de Montréal (IRCM), Montréal, Québec, Canada; ^4^Centre National de la Recherche Scientifique (CNRS), UMR7257, Université Aix-Marseille, France; ^5^Department of Biological Sciences, King Abdulaziz University, Jeddah, Saudi Arabia; ^6^Département de Microbiologie-Infectiologie et Immunologie, Faculté de Médecine, Université Laval, Québec, Québec, Canada; ^7^Present address: Department of Pharmacology, Dalhousie University, Halifax, NS, Canada

**Keywords:** phytopathogen, mating-type, cytochrome P450, CAZyme, terpene degradation

## Abstract

The ascomycete fungus *Ophiostoma novo-ulmi* is responsible for the pandemic of Dutch elm disease that has been ravaging Europe and North America for 50 years. We proceeded to annotate the genome of the *O. novo-ulmi* strain H327 that was sequenced in 2012. The 31.784-Mb nuclear genome (50.1% GC) is organized into 8 chromosomes containing a total of 8,640 protein-coding genes that we validated with RNA sequencing analysis. Approximately 53% of these genes have their closest match to *Grosmannia clavigera* kw1407, followed by 36% in other close Sordariomycetes, 5% in other Pezizomycotina, and surprisingly few (5%) orphans. A relatively small portion (∼3.4%) of the genome is occupied by repeat sequences; however, the mechanism of repeat-induced point mutation appears active in this genome. Approximately 76% of the proteins could be assigned functions using Gene Ontology analysis; we identified 311 carbohydrate-active enzymes, 48 cytochrome P450s, and 1,731 proteins potentially involved in pathogen–host interaction, along with 7 clusters of fungal secondary metabolites. Complementary mating-type locus sequencing, mating tests, and culturing in the presence of elm terpenes were conducted. Our analysis identified a specific genetic arsenal impacting the sexual and vegetative growth, phytopathogenicity, and signaling/plant–defense–degradation relationship between *O. novo-ulmi* and its elm host and insect vectors.

## Introduction

During the last centuries, increased movements of people and goods across countries and continents have favored the emergence and global spread of plant pathogens, insect pests, and invasive weeds which have substantially altered the landscape of several parts of the world. One well-documented example is Dutch elm disease (DED), the most destructive disease of elms. It has been estimated that over 1 billion mature elms were killed by two successive pandemics since the early 1900s (Paoletti et al. 2005). The first pandemic, which prompted initial investigations by Dutch scientists shortly after the First World War (Holmes and Heybroek 1990), was caused by the ascomycete fungus *Ophiostoma ulmi* (Buisman) Nannf. As it spread relentlessly over Western Europe and, a few decades later throughout North America, the disease caught the attention of both the general public and the plant pathology research community because of the devastation it brought to native elm populations. However, the second pandemic, which likely started around 1940–1950, was even more brutal than the first one as it was caused by a different fungus, *Ophiostoma novo-ulmi* Brasier, which was more virulent (used here as a quantitative attribute) and generally fitter than *O. ulmi*. Today, *O. novo-ulmi* is the dominant DED pathogen as it has almost totally displaced *O. ulmi* in most areas where DED is present. Two subspecies, *novo-ulmi* and *americana*, are recognized in *O. novo-ulmi* (Brasier and Kirk 2001). A third species, *Ophiostoma himal-ulmi*, occurs in the western Himalayas as an endophyte of native elm species which are highly resistant to DED (Brasier and Mehrotra 1995). Controlled inoculations of European elms with *O. himal-ulmi*, however, have confirmed that the latter is pathogenic to the more sensitive elm species and varieties found outside Asia (Brasier and Mehrotra 1995).

**Fig. 1.—:**
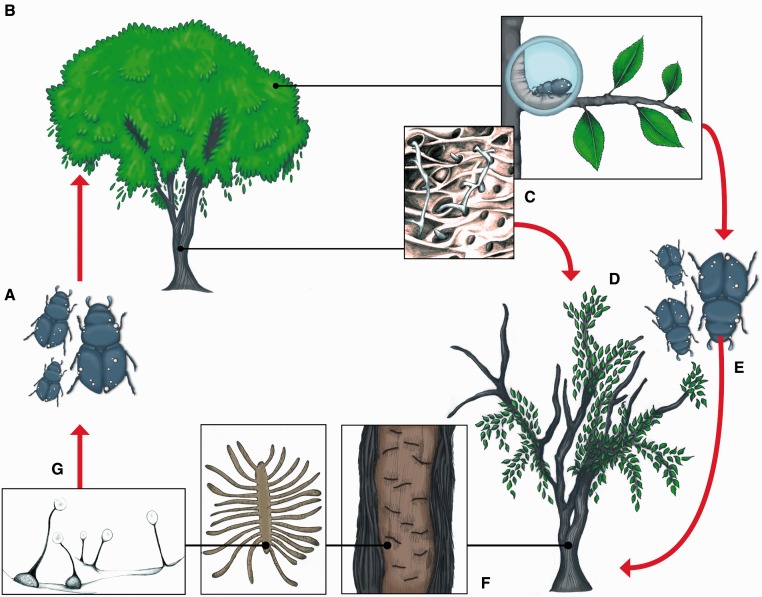
Disease cycle of DED. DED is caused by the exotic ascomycete fungi *O. ulmi*, *O. novo-ulmi,* and *O. himal-ulmi* which are vectored by elm bark beetles. In the absence of DED fungi, elm bark beetles complete their life cycle on elms without inducing any significant damage. Acquisition of *O. ulmi* and *O. novo-ulmi* by elm bark beetles, however, has had a catastrophic impact on elm species native to Europe and North America. When young elm bark beetles carrying spores of DED fungi (*A*) feed on healthy trees (*B*), they provide the pathogen with access to water-conducting vessels within the xylem. Invasion of the vascular system by DED fungi (*C*) rapidly induces wilting and eventually death (*D*). Trees that have been killed or weakened by DED attract virgin female bark beetles looking for suitable breeding sites. These females then emit aggregation pheromones that attract individuals of both sexes. Many of the beetles carry spores of DED fungi (*E*) and will thus allow the latter to colonize galleries in which females have oviposited after mating (*F*). There, the DED fungi produce abundant mycelium and reproductive structures (*G*) including elongated asexual synnemata and sexual perithecia with a globose base. Spores produced by both structures are embedded in a drop of sticky mucilage and will easily attach to the exoskeleton of young elm bark beetle adults (*A*) emerging from the galleries.

In nature, DED results from a complex, tripartite interaction between plants, scolytid insects, and pathogens (fig. 1). It begins when young adult bark beetles (*Scolytus* spp. and *Hylurgopinus rufipes*) move to the crown of healthy elms in late spring or early summer to feed on nutrient-rich phloem. Beetle feeding activity in the absence of the DED fungi is harmless to elms. However, if beetles carry spores of *O. ulmi* or *O. novo-ulmi* on their exoskeleton they allow the fungus to breach elm structural barriers (bark) and gain access to the xylem vascular system. Within xylem vessels, the fungus spreads by producing yeast-like budding spores and multicellular filamentous hyphae. The former allow passive vertical movement within individual vessels, whereas the latter enable the fungus to spread laterally and invade adjacent vessels through pit membranes. Development of the pathogen within elms induces a series of anatomical and physiological changes which, in susceptible species, culminates in death within a few weeks. Dead or moribund elms, in turn, emit volatile chemicals (kairomones) which attract virgin female elm bark beetles in search of suitable breeding sites. Female beetles then start excavating galleries in the inner bark and release aggregation pheromones that attract individuals of both sexes. Many of these individuals carry spores of DED fungi. Upon mating with a male, the female further constructs the gallery in which she will oviposit. After hatching from eggs, elm bark beetle larvae bore side-galleries perpendicular from the maternal gallery. Pupation takes place within the side-galleries. DED fungi grow saprophytically in galleries bored by female beetles and their progeny and colonize them extensively by producing a dense network of filamentous hyphae. It is also during this stage of their life cycle that DED fungi produce asexual fruiting bodies called synnemata which line the walls of the galleries and are topped by a droplet of sticky conidiospores embedded in mucilage. When sexually compatible individuals of DED-causing *Ophiostoma* spp. occur within galleries, perithecia will be formed which, like synnemata, exhibit the characteristic sticky droplets of spore-containing mucilage. Young elm bark beetle adults crawling out of the galleries come in contact with *Ophiostoma* fruiting bodies, acquire spores which attach to their exoskeleton, and eventually inoculate them to healthy elms when they feed. Webber (1990) reported that individual elm bark beetles emerging from the trunk and large branches of DED-killed elms could carry up to 350,000 spores of *O. ulmi*.

The DED pathogens are ophiostomatoid fungi, a polyphyletic complex of Ascomycete species associated with ambrosia and bark beetles. The main sexual genera currently recognized within this complex (Sordariomycetidae and Hypocreomycetidae) include *Ophiostoma*, *Grosmannia*, *Ceratocystiopsis,* and several new genera (including the original *Ceratocystis*) within the recently reorganized Ceratocystidaceae (de Beer et al. 2014). Most ophiostomatoid fungi described so far are saprobes of plants, but several species are known pathogens of agricultural crops and forest trees. In addition to *O. ulmi* and *O. novo-ulmi*, relevant examples include *Grosmannia clavigera* (a pathogen associated with massive mortality of lodgepole pine in western North America) and *Ceratocystis fimbriata* (a pathogen with a broad host range including deciduous trees and agricultural crops).

The first ophiostomatoid genome to be sequenced was that of *G. clavigera* kw1407 (DiGuistini et al. 2009, 2011). More recently, the genomes of *O. novo-ulmi* subsp. *novo-ulmi* H327 (Forgetta et al. 2013), *O. ulmi* W9 (Khoshraftar et al. 2013), *O. piceae* UAMH-11346 (Haridas et al. 2013), *C. fimbriata* CBS114723 (Wilken et al. 2013), *Ceratocystis manginecans* CMW17570 and *Ceratocystis moniliformis* CBS118127 (van der Nest et al. 2014), and *Sporothrix schenckii* ATCC58251 (Cuomo et al. 2014) were published. Of the published genomes, only that of *O. novo-ulmi* subsp. *novo-ulmi* strain H327 (thereafter referred to as H327) has been fully assembled into chromosome-length linear scaffolds. This was made possible by combining extensive conventional Roche/454 GS-FLX Titanium sequencing (6,425,848 reads; 2,529 Mb) with additional paired-end sequencing (181,162 reads; 7 kb average insert size). However, the genome of H327 was not annotated as the objective of the project was technical rather than biological: To test the reproducibility of the Roche/454 GS-FLX Titanium system across multiple core sequencing facilities (Forgetta et al. 2013).

Here, we report on the full biological annotation of the 31.784-Mb nuclear genome of H327 which is organized into eight chromosomes (chr). Among the over 8,000 protein-coding genes, we identified main components of H327’s genetic arsenal which control sexual and vegetative growth, phytopathogenicity, and the signaling/plant–defense–degradation relationship between *O. novo-ulmi* and its elm host and insect vectors.

## Materials and Methods

### Bioinformatic Analysis of the H327 Genome

#### Gene Calling and Initial Expressed Sequence Tag Alignment

Initial attempts at gene-calling were conducted with a local implementation of AUGUSTUS (using *Chaetomium globosum* and *Magnaporthe grisea* as example species; bioinf.uni-greifswald.de/augustus, last accessed December 19, 2014; Stanke and Waack 2003); however, this resulted in an artificial inflation of gene content by creating too many small coding sequences (CDS). A local implementation of GeneMark-ES (exon.gatech.edu, last accessed December 19, 2014; Ter-Hovhannisyan et al. 2008) generated a much more reasonable gene set (8,612 “raw” CDS; as reported by Forgetta et al. 2013) which only had to be slightly trimmed to remove spurious genes with total exon size of less than 100 bp (8,525 final “model” CDS). Previous banks of *O. novo-ulmi* expressed sequence tags (ESTs) (Jacobi et al. 2010; Hintz et al. 2011) were aligned to the H327 genome using BLAT (genome.ucsc.edu/FAQ/FAQblat, last accessed December 19, 2014) as implemented locally in a UCSC Genome Browser (genome.ucsc.edu/, last accessed December 19, 2014) and were used to initially verify the GeneMark-ES introns/exons. However, as their number was relatively modest and of uneven distribution (only ∼2,000 genes), we proceeded with high-throughput RNA sequencing (RNA-seq) for gene model correction (see below), resulting in a final count of 8,640 CDS.

#### Protein General Functional Analyses

General CDS annotations were done through local implementations of BLASTp (blast.ncbi.nlm.nih.gov; 10^−^^4^ *E* value cutoff) against GenBank nr (ftp://ftp.ncbi.nlm.nih.gov/blast/db, last accessed December 19, 2014), UniProt Knowledgebase and UniProt Swiss-Prot (www.uniprot.org, last accessed December 19, 2014) to achieve a manually verified consensus label for each CDS. Other manual protein domain verifications were conducted using Pfam (pfam.sanger.ac.uk, last accessed December 19, 2014). General functional (ontology) analyses (and statistics) were conducted with an online version of BLAST2GO Pro (www.blast2go.com, last accessed December 19, 2014) by importing the top ten hits from the above BLASTp analyses for each CDS (generated in XML format). Assignment of KEGG ontology was conducted using the KEGG Automatic Annotation Server (www.genome.jp/tools/kaas/, last accessed December 19, 2014; Moriya et al. 2007). Detailed annotations for all genes/proteins are presented in supplementary table S1, Supplementary Material online.

#### Noncoding RNAs, Repeat Elements, and Analysis of Repeat-Induced Point Mutation

Detection of transfer RNAs (tRNAs) was conducted using tRNAscan (lowelab.ucsc.edu/tRNAscan-SE, last accessed December 19, 2014; Schattner et al. 2005), rRNAs by RNAmmer (www.cbs.dtu.dk/services/RNAmmer, last accessed December 19, 2014; Lagesen et al. 2007), and general noncoding RNA characterization was conducted using Rfam (rfam.sanger.ac.uk, last accessed December 19, 2014). Repeat detection and characterization were done through local implementations of RepeatMasker ver. open-3.3.0 (www.repeatmasker.org, last accessed December 19, 2014) using RepBase update 2012-04-18 (www.girinst.org/repbase/update/index.html, last accessed December 19, 2014; Jurka et al. 2005) and through local BLASTn/manual analysis of regions of interest (high-AT regions, regions identified in dot-plots, etc.) to account for the addition of newer OPHIO and SWING elements not in the RepBase update. Analysis of repeat-induced point mutation (RIP) was conducted with a local implementation of RIPCAL (ripcal.sourceforge.net, last accessed December 19, 2014; Hane and Oliver 2008) or cumulative transition score (CTS) plots were computed as described in Bouvet et al. (2007). For the latter, RIPed copies were compared with the less-RIPed copy (internal reference) of the SWING retrotransposon and CpX → TpX and XpG → XpA transitions were scored as +1 and −1, respectively. The resulting curves represented the sum of all scores. An increasing slope denoted a preference for RIP on the matrix strand and, inversely, a decreasing slope denoted a preference for RIP on the complementary strand. As the number of RIP transitions increases so does the slope of the curve and vice versa. Finally, whole genome-to-genome alignments were conducted using WebACT (www.webact.org/WebACT/home, last accessed December 19, 2014) and within-genome dot-plots using Gepard (www.helmholtz-muenchen.de/icb/gepard, last accessed December 19, 2014; Krumsiek et al. 2007).

#### Carbohydrate-Active Enzymes, Cytochrome P450s, Peptidases, and Signal Peptides

Putative carbohydrate-active enzymes (CAZymes) were identified and assigned to families (and in some cases subfamilies) with the tools used for the daily updates of the CAZymes database (www.cazy.org, last accessed December 19, 2014; Lombard et al. 2014). Putative cytochrome P450s (CYP450s) were submitted to Dr David Nelson of the Cytochrome P450 Homepage (drnelson.uthsc.edu/CytochromeP450.html, last accessed December 19, 2014; Nelson 2009) for his manual verification and nomenclature assignments. Putative peptidases and exported proteins were identified using the online search tools of the MEROPS server (merops.sanger.ac.uk/index.shtml, last accessed December 19, 2014; Rawlings et al. 2012) and SignalP 4.1 server (www.cbs.dtu.dk/services/SignalP, last accessed December 19, 2014; Petersen et al. 2011), respectively.

#### Pathogen–Host Interaction and Secondary Metabolite Genes/Clusters

Genes potentially involved in host–pathogen interactions were identified using local BLASTp (*E* value cutoff of e^−^^20^) against the PHI-base curated database (www.phi-base.org, last accessed December 19, 2014; Winnenberg et al. 2008). Potential clusters of secondary metabolite genes were identified using the online SMURF tool (jcvi.org/smurf, last accessed December 19, 2014; Khaldi et al. 2010).

### Reverification of the H327 OPHIO3 DNA Sequence

The stand-alone copy of *OPHIO3* on the H327 chr.III was resequenced using the combinations of primers listed in supplementary table S2, Supplementary Material online, in order to obtain the complete 1,850-bp sequence. Total polymerase chain reaction (PCR) volumes were 50 µl containing: 1 × TaKaRa Premix *Ex Taq* (TaKaRa Bio), 0.2 µM of each primer (IDT DNA Technologies), and approximately 200 ng of template genomic DNA. Cycling conditions were an initial denaturation at 98 °C for 30 s, followed by 30 cycles of denaturation at 98 °C for 10 s, annealing at 55 °C for 30 s, extension at 72 °C for 2 min, and a final extension at 72 °C for 5 min. PCR products were Sanger-sequenced at the IBIS/Université Laval Plate-forme d’Analyses Génomiques. The sequence was not deposited in a public repository as it was found to be identical to the reference genome sequence generated by the recent pyrosequencing.

### Sequencing of the Mating-Type Locus from Additional Strains

In order to complete the mating-type analysis of strains related to H327, the entire mating-type locus was sequenced using the combinations of primers listed in supplementary table S2, Supplementary Material online, from the following strains: *O. ulmi* Q412T (MAT1-1), *O. novo-ulmi* subsp. *americana* VA (MAT1-1), *O. novo-ulmi* subsp. *americana* MH75 (MAT1-2), *O. novo-ulmi* subsp. *novo-ulmi* CKT11 (MAT1-2), *O. himal-ulmi* HP25 (MAT1-1), and *O. himal-ulmi* HP30 (MAT1-2). PCR reaction volumes and cycling conditions were as above, except the extension time for initial long-PCRs to generate the entire locus (∼8–10 kb) was 10 min. PCR products were then Sanger-sequenced as above and complete mating-type loci sequences were deposited in GenBank: KF961042–KF961047.

### H327 Self-Fertilization Mating Tests

The ability to self-fertilize by pseudoselfing (Brasier and Gibbs 1975) was assessed in *O. novo-ulmi* subsp. *novo-ulmi* H327 (MAT1-1) and CKT11 (MAT1-2), as well as in *O. novo-ulmi* subsp. *americana* VA (MAT1-1), FG245 (MAT1-1), and W2 (MAT1-2). Matings were carried out on elm sapwood agar supplemented with linoleic acid (ESAL; Bernier and Hubbes 1990). Each strain was tested in the following ways: 1) A yeast cell suspension (1,000 cells) was spread over ESAL; 2) two mycelium-bearing plugs were placed at 3 cm from each other on ESAL; and 3) a mycelium-bearing plug was placed at the center of an ESAL plate and the colony allowed to grow for 1 week before it was “fertilized” with a yeast cell suspension of the same strain. Each combination of strain and mating protocol was tested in triplicate. Positive mating controls were added in which strains carrying different *MAT1* alleles were plated together. Plates were inspected visually for the production of perithecia over 8 weeks.

### Cultivation of H327 in the Presence of Plant Terpenes

The response of yeast cells and mycelium of H327 to plant terpenes was initially tested in vitro. Yeast cells were grown in agitated flasks (120 rpm) containing either malt extract broth (Oxoid) or liquid minimal medium (MM) with proline as N source (Bernier and Hubbes 1990). Flasks were seeded with approximately 10^5^ cells ml^−^^1^ at the beginning of the experiments. Mycelia were grown on plates of malt extract agar (MEA; Oxoid) or MM with ammonium sulfate as N source (Bernier and Hubbes 1990). Inoculum consisted of a mycelium-covered plug of solid medium placed at the center of each Petri dish. Alpha-pinene, β-pinene, and limonene (Sigma) were tested individually at concentrations ranging from 0.05% to 1.0% v/v with 2–5 replicates. Yeast cell growth kinetics in liquid media and mycelial growth rate on solid media were assessed over 14 and 7 days, respectively. All experiments included controls without terpenes.

For subsequent quantitative PCR (qPCR) experiments, isolate H327 was initially inoculated on solid MEA medium and incubated at 22–24 °C. For liquid cultures (yeasts), disks of these solid cultures were inoculated into liquid MEA and incubated at 22–24 °C with agitation (110 rpm). After 3 days, the 50 ml liquid culture was split into 4 × 10 ml subcultures containing 0.1% v/v α-pinene, β-pinene, limonene, or an unamended control. For solid cultures (mycelium), disks of H327 were transferred onto sterilized cellophane circles (∼7–8 cm ø) on MEA plates and incubated at 22–24 °C for 3 days. Three membranes per treatment were transferred to fresh MEA plates containing the same concentrations of terpenes as listed above. Incubation of both types of cultures then continued as above until harvesting at *t* = 18, 24, and 48 h postexposure to terpenes. Liquid cultures were harvested by subsampling 1 ml from each tube, followed by centrifugation at 3,200 × *g* for 4 min, discarding the supernatant and then snap freezing with liquid nitrogen. Solid cultures were harvested by scraping the mycelial growth from half or the entire membranes (depending on biomass) into Eppendorf tubes then snap freezing with liquid nitrogen. The plates were then discarded after sampling; hence one was prepared per time-point. Total RNAs were then extracted from the samples using tungsten carbide beads and a laboratory mixer mill (Mixer Mill MM300, Retsch Inc.) for tissue disruption, followed by purification using the Plant RNeasy Kit (QIAGEN) and quantification on a NanoDrop ND1000 (Thermo Scientific). One-hundred nanograms of pure RNA per sample were converted to cDNA using the High Capacity cDNA Reverse Transcription Kit (Invitrogen-Life Technologies) according to the manufacturer’s instructions, with the exchange of the supplied random primer for an oligo dT_18_ at 5 μM final concentration.

### qPCR of CYP450 Expression

Transcript numbers of *g7466* (CYP52P6—putative terpene degradation) and *g2373* (“control” CYP51F1—membrane ergosterols) were determined using an Applied Biosystems 7500 Fast qPCR system (Life Technologies) with PerfeCTa SYBR Green FastMix Kits (Quanta BioSciences) on the above cultures grown in the presence of terpenes. Novel qPCR primers (supplementary table S2, Supplementary Material online) were designed to produce small (∼280 bp), efficiently amplified products. Assays were optimized using annealing gradients and specificities were checked through melting curve analyses. The 20 µl reactions contained: 10 µl 2 × PerfeCTa mix, 0.3 µM (final concentration) of each primer, 5 µl of cDNA template dilutions, and q.s. Milli-Q water. Triplicate (at least) reactions for each sample were run. Quantification was achieved relative to known PCR product standards using 10-fold dilution series ranging from 10^9^ to 10^2^ copies. The standards were 1,000–1,020 bp purified CYP gene products generated from H327 DNA using the primers listed in supplementary table S2, Supplementary Material online. Standard curve *r*^2^ values were ≥ 0.99 and sample efficiencies averaged 88% for both targets combined.

### RNA-seq Analysis

Using techniques similar to those listed above, yeast- (liquid) and mycelial-form (solid) H327 cultures grown in/on complete medium (Bernier and Hubbes 1990) were harvested after 5 days growth, RNAs were extracted using the RNeasy Mini Kit (QIAGEN; yeast protocol) and nucleic acids were quality controlled using the NanoDrop and BioAnalyzer RNA 6000 Nano Kit (Agilent Technologies). mRNA-focused cDNA libraries were synthesized using the TruSeq RNA Sample Preparation Kit v2 (Illumina) using 1 µg of starting material at the IBIS/Université Laval Plate-forme d’Analyses Génomiques. Libraries were sequenced on one lane of an Illumina HiSeq 2500 (v1.9 single-end 100 bp) at the McGill University and Génome Québec Innovation Centre. The two samples (yeast + mycelial) utilized herein for gene correction were part of 12 bar-coded samples run on the same above lane that will be the subject of a forthcoming study on H327 dimorphism expression profiling (Nigg M and Bernier L, unpublished data). The raw RNA-seq data were submitted to the NCBI Sequence Read Archive under accession SRP047075.

The approximately 28 million raw reads (supplementary table S3, Supplementary Material online) were filtered/quality-controlled using FastQC v0.11.2 (www.bioinformatics.babraham.ac.uk/projects/fastqc/, last accessed December 19, 2014) and Prinseq v0.20.4 (prinseq.sourceforge.net/, last accessed December 19, 2014) as follows: Adaptors and poly-A/T tails (>10 nt) were trimmed; sequences with greater than 20% Ns, less than 20 nt in length and exact duplicates were removed completely. We then used both Newbler v2.8 (GS de novo Assembler; Roche) and TopHat v2.0.10 (ccb.jhu.edu/software/tophat/index.shtml, last accessed December 19, 2014; Kim et al. 2013) aligners to control for individual software variability in mapping quality. Once mapped, TopHat output BAM files and a GTF of the “final model” CDS from above were fed into the Artemis genome browser and annotation tool (www.sanger.ac.uk/resources/software/artemis/, last accessed December 19, 2014; Carver et al. 2012) for manual correction of the entire gene set. In the case of alternatively spliced transcripts, the most common variant (or longest if similar frequencies) for any one gene was selected for the final gene set. The final validated gene, transcript, and protein sets are available as supplementary datafiles S1–S3, Supplementary Material online.

## Results and Discussion

### Overall Organization and Gene Content

**Fig. 2.—:**
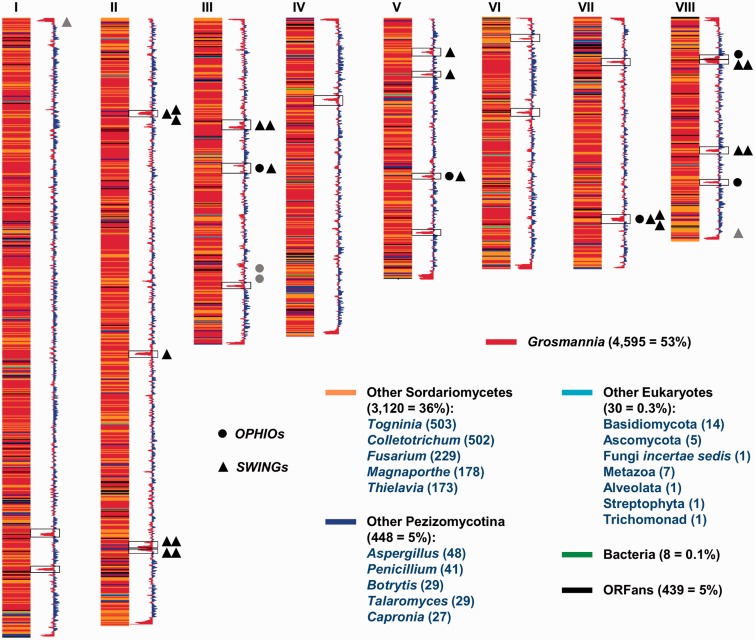
Representation of the *O. novo-ulmi* H327 chromosomes and GC content. The chromosomes are represented by stacks of vertical strips, one for each gene, which are colored according to the first BLAST match against the nr database in GenBank (using BLASTp of the resulting proteins), excluding the very close *Ophiostoma*/*Sporothrix* sister species. The height of each gene strip is proportional to the gene size, but intergenic spaces are ignored. For each category/lineage of source hits, the numbers in parentheses indicate the number and percent of proteins out of the 8,640 total matching the group. For the two fungal categories, the numbers of hits to the top five genera are shown; and for the Other Eukaryotes category, all 7 taxa comprising the 30 hits are shown. ORFans indicate those proteins with no BLASTp matches at an *E* value cutoff of 10^−4^; however note that the real number is only 181 once the *Ophiostoma*/*Sporothrix* sister species are readded (but still excluding *O. novo-ulmi* self-hits). Adjacent to the chromosomes are the vertical GC content graphs, wherein values above average are colored blue and below average colored red. The 22 regions of excessively high AT (excluding telomeres) are surrounded by rectangles. The approximate locations of the *OPHIO* elements (circles; fig. 4) and *SWING* elements (triangles; fig. 5) are indicated, with those associated with the high-AT regions in black versus those (only 4 = 2 *OPHIO* + 2 *SWING*) elsewhere in gray.

**Fig. 3.—:**
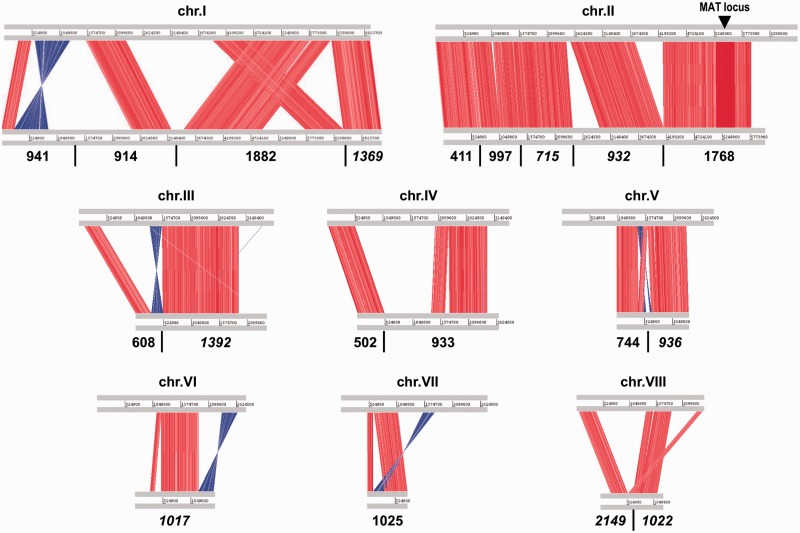
WebACT genome-to-genome comparison of *O. novo-ulmi* H327 versus *O. ulmi* W9. H327 chromosomes (top gray bars) are aligned to W9 scaffolds (bottom gray bars). For simplicity, only W9 scaffolds greater than 0.5 Mb were aligned (those in italics reverse complemented), therefore some gaps are actually filled with (multiple) very small contigs. These 19 scaffolds represent 23.2 Mb of the 31.5 Mb genome (∼74%). The vertical red/blue bars between the sequences indicate ≥80% nucleotide identity. Note the large region (∼360 kb) of higher-than-average identity (>95%) around the MAT locus between both genomes (detailed in supplementary fig. S6, Supplementary Material online).

The H327 genome is similar in size (31.8 Mb) to the genome of the pine phytopathogen *G. clavigera* kw1407, the closest assembled ophiostomatoid fungus (DiGuistini et al. 2011), but slightly smaller than other assembled genomes from Sordariomycete fungi; however, H327 is the only one with an extra chromosome (table 1). The eight H327 chromosomes, varying from approximately 2.5 to 7 Mb each (fig. 2 and supplementary table S4, Supplementary Material online), concord well with what has been observed in PFGE analysis (Et-Touil et al. 1999; Bernier 2013). The recently sequenced *O. ulmi* W9 (Khoshraftar et al. 2013) and *O. piceae* UAMH-11346 (Haridas et al. 2013) are very similar in size (31.5 and 32.8 Mb) to H327, but their genomes are not assembled and we can therefore make no conclusions as to their comparative karyotypes. However, we were able to do a preliminary comparative analysis between H327 and the largest *O. ulmi* W9 scaffolds showing significant chromosomal indels and rearrangements between the two (fig. 3), even though they share fairly high nucleotide similarity. This is not necessarily unexpected as previous PFGE results have shown different strains of *Ophiostoma* can be isolated with large chromosomal rearrangements, including gains or losses of chromosomes through recombination (Dewar and Bernier 1995; Dewar et al. 1997).

**Table 1:** Genome Statistics for *Ophiostoma novo-ulmi* H327 Compared with Other Closely Related, Sequenced, and Assembled Fungal Genomes

	Sordariomycetes				
	Ophiostomatales				
Characteristic	*Ophiostoma novo-ulmi* H327	*Grosmannia clavigera* kw1407	*Magnaporthe orzyae* 70-15	*Neurospora crassa* OR74A	*Podospora anserina* S mat +
Genome size (Mb)	31.8	29.8	41.7	41.0	∼36
Number of chromosomes	8	7	7	7	7
%GC genome	50.1	53.4	51.6	48.3	52.0
%GC transcripts	57.0	60.5	56.0	54.0	55.9
%GC exons	57.9	nr	57.3	54.6	nr
Protein-coding genes	8,640	8,314	11,043	9,733	10,545
Percent coding	45.3	45.8	46.4	48.0	44.8
Gene density	1 per 3.7 kb	1 per 3.5 kb	1 per 3.8 kb	1 per 4.2 kb	1 per 3.4 kb
Mean intergenic distance (bp)	1,842	1,466	2,041	2,297	nr
Mean gene length (bp)	1,786	1,641	1,754	2,023	nr
Mean protein length	555 aa	545 aa	481 aa	486 aa	496 aa
Percent genes with introns	59.0	77.2	84.8	91.6	nr
Mean number of introns/gene	1.77	1.86	nr	1.7	1.27
Mean intron length (bp)	114	70 ^a^	Nr	134	79
tRNA genes	254	nr	341	415	361
5 S rRNA genes	43	nr	46	79	87

Note.—Statistics were compiled from the original genome publications, with modifications as per the most current assemblies available in public databases: For *Grosmannia*, DiGuistini et al. (2011); for *Magnaporthe*, Dean et al. (2005) and the Broad Institute (www.broadinstitute.org/annotation/genome/magnaporthe_grisea/MultiHome.html, last accessed December 19, 2014); for *Neurospora*, Galagan et al. (2003) and the Broad Institute (www.broadinstitute.org/annotation/genome/neurospora/MultiHome.html, last accessed December 19, 2014); and for *Podospora*, Espagne et al. (2008). nr, not reported in the associated publications, nor calculable from publicly available information.

^a^Only the median intron length, and not mean, was reported/available.

Each H327 chromosome codes for 667–1,867 proteins, for a total of 8,640 proteins, giving H327 a similar coding density to its close relatives (table 1). Excluding the very close *Ophiostoma*/*Sporothrix* sister species, approximately 53% of the H327 proteins have their closest match to *G. clavigera*, followed by 36% in other close Sordariomycetes, 5% in other Pezizomycotina, and surprisingly few (439 = 5%) orphans (ORFs with no known homologs—actually only 181 once the *Ophiostoma*/*Sporothrix* sister species are readded) (fig. 2 and supplementary figs. S1 and S2, Supplementary Material online). The proteins were confirmed through our RNA-seq analysis which adopted a redundant approach to read-mapping by employing Newbler and TopHat aligners (see Materials and Methods) to account for software variability in mapping quality. In our case, TopHat achieved superior performance and showed that 99% (8,521/8,640) of the final gene models could be detected with at least one read (supplementary table S3, Supplementary Material online). Nearly 90% of genes were also covered by at least 20 reads which, given our average read length of 96 nt and the average mature transcript length of 1,665 nt, essentially correspond to 1-fold coverage along the entire length of the average gene. The approximately 30% of reads mapped to the whole genome which did not match exon + intron regions represented pseudogenes, remains of repetitive elements (REs) (common in the high-AT regions) and, primarily, 5′/3′-UTRs that can be of significant sizes. Manual correction of the entire gene set showed that initial in silico predictions were of good quality—less than 1% of genes were spurious and only approximately 2% were missing (supplementary table S5, Supplementary Material online), the majority of which were “created” from gene splits from incorrect gene fusions and not from completely novel gene discovery (only 25 genes). However, the vast majority of corrections (still only ∼9% of all genes) involved existing genes with incorrect internal (exon–intron) or external (start/stop) boundaries, with the most common error being the incorrect 5′-extension of genes in order to encompass very distant spurious start codons. A small proportion (147 = 1.7%) of genes presented significant alternative splicing (supplementary table S1, Supplementary Material online) and will have to be investigated further to elucidate their differential expression. Overall, we have been able to significantly increase our transcriptomic information for H327 (∼8,500 genes) from previous banks of ESTs (∼2,000 genes; Jacobi et al. 2010; Hintz et al. 2011) and have achieved higher coverage/gene model detection (∼99%) than other close organisms (e.g., ∼80% for *O. ulmi* [Khoshraftar et al. 2013] and 92% for *G. clavigera* [DiGuistini et al. 2011]) from only two banks from axenic cultures.

### Telomeres, Repeat Regions, and Mobile Elements

The H327 telomeres vary from 17 to 160 bp in size (probably inaccurate due to repeat handling in assemblers) and are made up of a cumulative 10 × TTAGGG + 77 × TTAGG + 25 × TTAG motifs, arranged in random combinations within each telomere. The majority TTAGG motif with only two G’s is apparently unique compared with the telomere consensus sequence (TTAGGG) in other close ascomycetes (Cohn et al. 2005); however, this may be an artifact of pyrosequencing which has difficulty with homopolymers.

Telomeres included, a relatively small portion (∼3.4%) of the H327 genome is occupied by repeat sequences (table 2); however, this may be explained by the presence of RIP. RIP is a mechanism, present in filamentous ascomycetes, that inactivates duplicated genomic DNA during sexual reproduction in order to prevent the uncontrolled spread of mobile/repeat elements (REs; Clutterbuck 2011). RIP preferentially mutates C:G pairs to A:T (often affecting CpA dinucleotides the most), thereby increasing the %AT of the regions targeted for inactivation. The H327 genome has 22 regions of excessively high AT (mean 73%, range 65–79%; fig. 2) which show the scars of concerted RIP action. The in silico gene callers indicated many degraded genes/pseudogenes in these regions and careful examination of these fragments showed that they belonged to various classes of REs that have been inactivated by the introduction of multiple stop codons. Many of the 22 regions also show nucleotide-level homology to one-another (supplementary fig. S3, Supplementary Material online), with many duplications and inversions, indicating that they were often copied into themselves or similar REs that subsequently were the targets of RIP. The sequenced genomes of the pine and rice phytopathogens *Dothistroma septosporum* (de Wit et al. 2012) and *M. grisea* (Dean et al. 2005) show similarly strong clustering of REs in high-AT regions which have been degraded by less-severe RIP action compared with the model fungus *Neurospora crassa* (where 97% of repeats > 400 bp are RIPed; Galagan et al. 2003).

**Table 2:** REs in the *Ophiostoma novo-ulmi* H327 Genome

Type of Element	Number	Total Length (bp)	% Genome
Retroelements	1,602	429,087	1.35
SINEs	25	1,416	<0.01
LINEs	746	81,416	0.26
LTRs	831	346,255	1.09
DNA transposons	400	47,147	0.15
Unclassified	37	2,698	<0.01
Total interspersed repeats	2,039	478,932	1.51
Satellites	25	2,226	<0.01
Simple repeats	3,348	178,359	0.56
Low Complexity	6,651	413,517	1.30
Total all repeats	12,063	1,073,034	3.38

Note.—Compiled from statistics generated by RepeatMasker/RepBase (www.girinst.org/repbase/, last accessed December 19, 2014). SINE, short interspersed element; LINE, long interspersed element; LTR, long terminal repeat element.

**Fig. 4.—:**
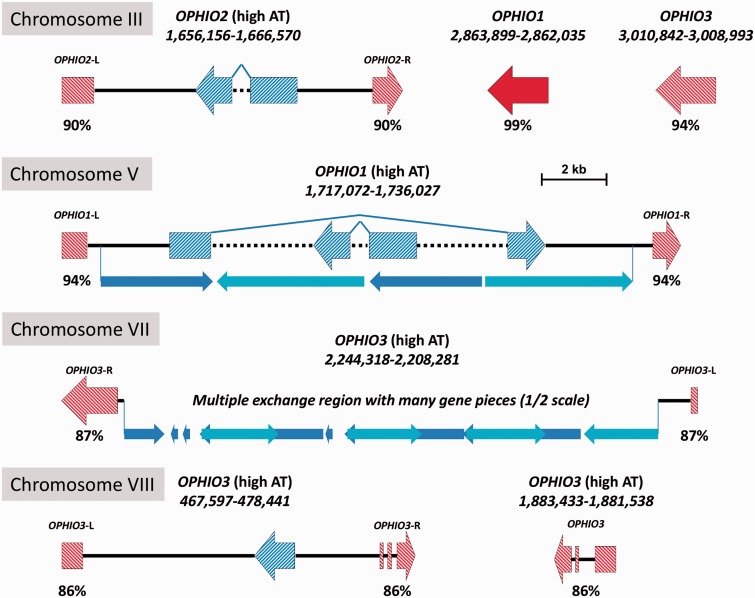
Locations, genomic contexts, and functionalities of the *OPHIO* DNA transposon REs in the *O. novo-ulmi* H327 genome. *OPHIO* elements are represented by red block arrows and genes by blue block arrows. The still-functional elements/genes (*OPHIO1* on chr.III and *g8426* on chr.VIII) are marked by solid colors and nonfunctional elements/genes (possessing many stop codons) by hash patterns. Solid black lines indicate intergenic, nontranscribed space, whereas dashed lines indicate (relic) introns. Only two *OPHIO* elements are stand-alone and contiguous (one functional), whereas the others are fragmented into multiple pieces by intervening high-AT-content DNA. Note that the intervening DNAs in two of the *OPHIO* elements (chr.V and VII) show many duplications and rearrangements and their physical layouts (determined by dot-plot) are indicated by the small light/dark blue arrows below the main lines. Also note that the region on chr.VII is at half-scale due to its large size and shows multiple inverted repeats, hence the overlaps in certain sections of both colors.

As a first example of the effects of the RIP mechanism, we present in figure 4 the inventory and genome contexts of the OPHIO-type DNA transposons in the H327 genome. These type II elements were first discovered in *O. novo-ulmi* and *O. ulmi* (Bouvet et al. 2007), and so far appear to be *Ophiostoma*-specific. H327 contains seven copies of all three different OPHIOs (*OPHIO1*, *OPHIO2,* and *OPHIO3*), five degraded/nonfunctional copies of which are associated with five of the high-AT regions where they have been split by intervening DNA (all but one). Stand-alone copies of *OPHIO1* and *OHPIO3* are present, but *OPHIO1* is the only element whose bioinformatic and experimental analysis (Bouvet et al. 2008) shows it is still functional. All of the nonfunctional OPHIO elements show strong evidence of typical RIP signatures (including a slight preference for mutating CpA over CpT; supplementary fig. S4 and table S6, Supplementary Material online). We discovered some sequence discrepancies (*n* = 93) between the chr.III copy of *OPHIO3* compared with the original sequencing of Bouvet et al. (2007) in the same strain. We resequenced the full-length element through the traditional Sanger method using the same DNA we used for pyrosequencing and verified that our two sequences were identical. This indicates that either the sequencing of Bouvet et al. (2007) inadvertently contained errors or there is yet another stand-alone copy of *OPHIO3* somewhere else in the H327 genome within the small amount of gaps (∼84 kb) in our assembly. There is curiously only one copy of *OPHIO2*, which should have excluded it from RIP, indicating potentially the same scenario of a “hidden” copy, supported by the original southern hybridizations by Bouvet et al. (2007) which suggested multiple copies of *OPHIO2* in H327. Regardless of whether OPHIO elements are present or not within the 22 AT-rich regions, relics of other REs are and the regions all clearly show evidence of RIP through their dinucleotide frequencies (supplementary fig. S4, Supplementary Material online).

**Fig. 5.—:**
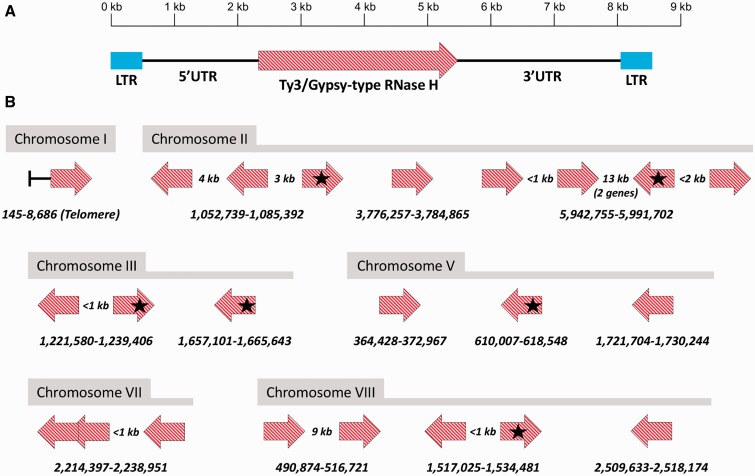
Schematic and locations of SWING retrotransposons in the *O. novo-ulmi* H327 genome. (*A*) The approximately 8.5-kb type I elements have approximately 490-bp LTRs (blue) on each end and contain a nonfunctional Ty3/Gypsy-type RNase H gene of a probable new family. UTR, untranslated region. (*B*) Genomic locations and contexts of SWINGs with colors and patterns as in fig. 4, except all elements are in high-AT regions unless otherwise indicated and those associated with gene transcription start sites (within ∼2 kb upstream) are marked with black stars. Note that, unlike in part (*A*), the arrows in (*B*) represent the whole elements (RNase + UTRs + LTRs).

As a second example of RIP, and in order to demonstrate that the phenomenon is general in nature and not just targeted to the *OPHIO* type II REs, we analyzed a new type of retrotransposon discovered during the analysis of the H327 genome. We call these approximately 8.5 kb type I elements SWINGs—they have approximately 490-bp long terminal repeats (LTRs) on either ends (fig. 5) and encode Ty3/Gypsy-type RNase H, although identity is low (∼40% protein level to closely related Ascomycetes) with other REs indicating the SWING elements form their own new “family” according to the classification scheme of Wicker et al. (2007). SWINGs also appear to be H327-specific so far, as a search of the most closely related *O. ulmi* W9 genome showed only a few copies of the LTR present on a few of the scaffolds, but never in pairs with intervening DNA that could code for the whole mobile element. There are 23 stand-alone/whole copies of the SWING retrotransposon spread throughout six of the eight chromosomes—all but two are associated with the high-AT regions, similar to the OPHIO REs above. Even though chr.IV and VI do not have any whole copies, all chromosomes show dozens of pieces of SWINGs each, concentrated in the high-AT regions. All of these pieces show signs of degradation by RIP and, by using CTSs (another way of visualizing/quantifying the impact of RIP; Bouvet et al. 2007), it is clear that even the stand-alone copies are all severely RIPed (supplementary fig. S5, Supplementary Material online). Six SWINGs (∼25%) are located in promoter regions (between roughly −2,000 nt and the transcription start site) which could influence gene expression; two of the genes are unknowns, one is an amino acid transporter, and the remaining three code for enzymes involved in basal metabolism of amino acids. The emergent idea that transposable elements act as a rapid evolutionary mechanism to wire up genomic regulatory networks is now well accepted (Ellison and Bachtrog 2013) and is known as exaptation or “domestication” of REs into novel *cis*-regulatory elements (de Souza et al. 2013; Riordan and Dupuy 2013). As SWING elements are not present in other *Ophiostoma* spp., we could hypothesize that SWING copies may have evolutionary consequences and drive certain aspects of pathogenicity specifically in *O. novo-ulmi*.

Curiously, despite all the strongly RIPed sequences in H327, the *RID* gene (for RIP-deficient upon mutation) responsible for the RIP mechanism does not appear to be present in the H327 assembly. With the exception of those within the Saccharomycotina and Taphrinomycotina, only three ascomycete fungi do not have the *RID* gene out of the greater than 60 whose genomes have been examined: *Blumeria graminis*, *G. clavigera* (closest to *O. novo-ulmi*), and now *O. novo-ulmi* subsp. *novo-ulmi* H327 (Malagnac F, personal communication). The data from Bouvet et al. (2007) suggest that RIP may not be functional in *O. novo-ulmi* subspecies *americana*, perhaps pointing to its lack of a *RID* gene as well, and the recent *O. ulmi* genome assembly (3,415 contigs) also does not have a significant *RID* homolog. The authors do not discuss the status of RIP within this latter genome, but our analysis concurs with Bouvet et al. (2007) that it is (or was recently) functional, as some OPHIO elements contain signature RIP dinucleotide changes. Taken together, these data suggest either 1) a recent loss of RIP function (through loss of the *RID* gene) in the common ancestor of *O. novo-ulmi* and *O. ulmi*, not leaving enough time for random mutation to have erased the RIP signatures; 2) an as-yet unidentified functional analog of *RID* is maintaining RIP function in these genomes; or 3) as for some of the REs above, the *RID* gene may be present in the small assembly gaps. Further investigation of the topic is required, perhaps by following duplicated marker genes in H327 for signs of RIP after multiple rounds of sexual reproduction.

### General Identifiable Functions

#### Overview

**Fig. 6.—:**
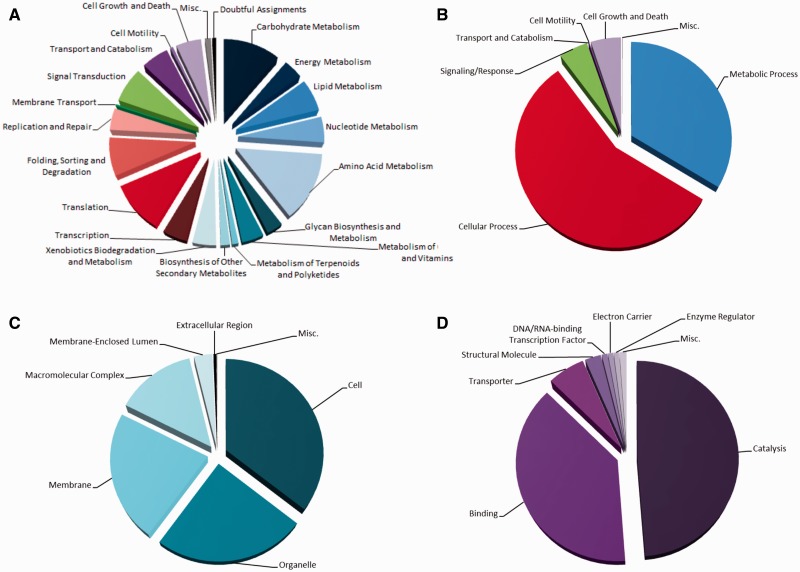
Functional distributions of *O. novo-ulmi* H327 proteins. Classification of protein functions by (*A*) KEGG or (*B–D*) BLAST2GO (Gene Ontology). The latter categorizes proteins in three different ways—biological process (*B*; concords with KEGG), cellular component (*C*), and molecular function (*D*).

Although the KEGG system was able to place many of the H327 proteins into a multitude of metabolic pathways, its overall performance was relatively poor, being able to assign a KEGG number to only approximately 36% of the proteins (table 3). Their detailed functions were divided as follows: Approximately half involved in metabolic functions (metabolism/biosynthesis), one-quarter in cellular processes (translation, replication, etc.), and significant-sized blocks in signaling, transport and cell growth/death (fig. 6*A*). Much better performance was obtained using Gene Ontology analysis (BLAST2GO): Approximately 76% of the proteins could be assigned functions and their overall distributions were similar to KEGG, although with more emphasis on cellular processes (fig. 6*B*–*D*). The SignalP algorithm also allowed us to postulate that approximately 7% of the total protein complement in H327 is destined for export (table 3), a nearly identical proportion when compared with other necrotrophs such as *G. clavigera*, *Botrytis cinerea,* and *Sclerotinia sclerotiorum* (Amselem et al. 2011; DiGuistini et al. 2011).

**Table 3:** Summary of Protein Types/Clusters in the *Ophiostoma novo-ulmi* H327 Genome

Characteristic	Tool/Database	Number	Percent
General function/homology			
Proteins with at least 1 homolog (nonself)	BLASTp	8,459	97.9
Proteins assigned at least 1 GO term	BLAST2GO	6,538	75.7
Proteins assigned a KEGG number	KEGG Mapper	3,073	35.6
Proteins with signal peptides	SignalP	621	7.2
Peptidases and Inhibitors (no. secreted)	MEROPS	295 (56)	3.4
Proteins assigned to a CAZy family (no. secreted)	CAZy	311 (115)	3.6
Glycoside hydrolases—GT		163 (93)	1.9
Glycosyltransferases—GH		79 (5)	0.9
Polysaccharide lyases—PL		2 (2)	<0.1
Carbohydrate esterases—CE		20 (9)	0.2
Carbohydrate-binding modules—CBM		35 (16)	0.4
Auxiliary activities—AA		12 (—)	0.1
Cytochrome P450s	CYP Homepage	48	0.6
Number of unique families		39	—
Families shared with *G. clavigera* kw1407		26	—
Families different from *G. clavigera* kw1407		13	—
Proteins involved in pathogen–host interactions	PHI-base	1,731	20.0
Unique PHI-base numbers/proteins		1,051	—
Mutants have increased virulence or antagonism		13	0.2
Mutants are unaffected in pathogenicity		680	7.9
Mutants have reduced virulence		701	8.1
Mutants lose pathogenicity		149	1.7
Mutants are lethal		82	0.9
Mutants show mixed results		88	1.0
Effector (plant avirulence determinant)		4	<0.1
Chemistry target (phenotype unknown)		14	0.2
Fungal secondary metabolite clusters	SMURF	7	—
“Backbone” genes (PKS and like)		9	0.1
“Backbone” genes (NRPS and like)		2	<0.1
Range of genes in clusters		2–18	—
Total number of genes in all clusters		67	0.8

Note.—BLASTp, Protein BLAST at NCBI (blast.ncbi.nlm.nih.gov, last accessed December 19, 2014); BLAST2GO, Blast to Gene Ontology (GO) (www.blast2go.com, last accessed December 19, 2014); CAZy, Carbohydrate-Active Enzyme Database (www.cazy.org, last accessed December 19, 2014; manual annotation by B. Henrissat); CYP Homepage, Cytochrome P450 Homepage (drnelson.uthsc.edu/CytochromeP450.html, last accessed December 19, 2014; manual annotation by DR Nelson); KEGG Mapper, Kyoto Encyclopedia of Genes and Genomes Mapper (www.genome.jp/kegg/mapper.html, last accessed December 19, 2014); MEROPS Peptidase Database (merops.sanger.ac.uk, last accessed December 19, 2014); PHI-base, Pathogen–Host Interactions Database (www.phi-base.org, last accessed December 19, 2014); SignalP, Signal Peptide Cleavage Site Predictor (www.cbs.dtu.dk/services/SignalP, last accessed December 19, 2014); SMURF, Secondary Metabolite Unique Regions Finder (jcvi.org/smurf, last accessed December 19, 2014).

#### Carbohydrate Enzymes

Using the CAZy system described in Cantarel et al. (2009), we were able to identify 311 genes encoding potential CAZymes in the H327 genome (table 3 and supplementary table S7, Supplementary Material online). The profiles of the different CAZyme families present in H327 and the closely related *G. clavigera* genome are fairly overlapping, with similar major GH/GT/CBM classes, although H327 has slightly more (20 vs. 7) carbohydrate esterases which remove ester-based modifications from complex polysaccharides. Compared with the CAZyme complement in other Pezizomycotina, H327 has a much smaller complement overall, even more so than would be expected just due to its smaller genome size. Specifically, whereas some CAZyme families are relatively abundant in all members of the group (e.g., families GH3, GH5, GH16, GH18, GT2), H327 is lacking in others (e.g., families GH13, GH43, CBM1, CBM18, and AA9). Interpreting the profiles at a finer level of differential substrate/target specificities is not always straightforward—although we have included the putative targets for the CAZymes in supplementary table S7, Supplementary Material online, the family-to-function relationships are often difficult to assess, as sequence similarity (which assigns the family designation) can group proteins with varying specificities (Stam et al. 2006; Aspeborg et al. 2012). That being said, part of the difference may be explained by the fact that many of the other sequenced members of the Pezizomycotina are saprophytic and there is a general expectation that these fungi will have more developed CAZyme panels (Amselem et al. 2011); however, the relationship does not hold in all cases (supplementary table S7, Supplementary Material online). Perhaps more determinative, *O. novo-ulmi* gains direct access to the elm vascular system through its vector and it therefore does not have to penetrate outer plant layers (bark, epidermis, etc.), unlike other vascular pathogens such as *Fusarium oxysporum* and *Verticillium dahliae*.

#### Cytochromes

Forty-eight CYP450s (one potential pseudogene) were identified in the genome and classified into 39 unique families according to the nomenclature established by Nelson (2006), 13 of which are unique to H327 compared with *G. clavigera* (table 3 and supplementary table S8, Supplementary Material online; the latter has 17 unique compared with H327). The largest groups of CYP450s with similar putative functions are 9 involved with pisatin (plant defense), 7 with benzene/phenolics (plant defenses), 6 with sterigmatocystin (ST) (mycotoxin), and 3 with trichothecenes (mycotoxins); covering approximately half of H327’s entire “CYPome.” As with the CAZymes above, care must be taken when using sequence similarity as small amino acid changes can translate to large specificity changes; however, we have attempted to assign putative functions (or general pathway involvements) for the various CYP450s (supplementary table S8, Supplementary Material online) as in Kelly et al. (2009) for *Aspergillus* and Lah et al. (2013) for *Grosmannia* (which includes RNA-seq information). The three large groups, along with a single CYP450 putatively involved in terpene degradation and another single CYP450 present within a secondary metabolite cluster, will be discussed below in the context of phytopathogenicity and vector interactions.

#### Peptidases

Approximately 300 peptidases (in ∼60 families) were identified in H327 according to the MEROPS system (Rawlings et al. 2012; table 3), comparable to the quantity in *G. clavigera* (DiGuistini et al. 2011). Examining the 56 exported peptidases more closely can be informative as these hydrolytic enzymes are implicated not only in nutrition but also in the degradation of host plant tissues and proteinaceous defense molecules (Monod et al. 2002; Olivieri et al. 2002). First, 26 of them are best suited for activity in acidic conditions versus 14 in basic conditions (remainder neutral pH), implying that acidic environments may be more frequently encountered. There was a similar “acid preference” in secreted peptidases from the broad-host-range phytopathogenic fungi *B. cinerea* and *S. sclerotiorum* (Amselem et al. 2011). American elm (*Ulmus americana*) sap and water extracted from wood are known to be slightly acidic (pH 6.0–6.5; Hartley et al. 1961) and *O. novo-ulmi* shows maximum growth rates in liquid cultures between pH 5.8–6.8 (Harris and Taber 1970). One may also hypothesize that the galleries formed by the bark beetles (*H. rufipes* and *Scolytus* spp.) serving as vectors for *O. novo-ulmi* may tend toward an acidic pH. Second, although the majority has broad activities mainly associated with nutrition (such as 12 pepsins, 7 subtilisins, and 7 sedolisins [families A0, S08, and S53]), six peptidases (families S28 and S33) are only active on terminal prolines and seven others (family S09) are active mostly only on small oligopeptides. These two classes of exported peptidases can possibly strongly affect fungus–elm interactions and will be more thoroughly discussed in the context of phytopathogenicity below.

### Sexual and Vegetative Growth Genes/Pathways

**Fig. 7.—:**
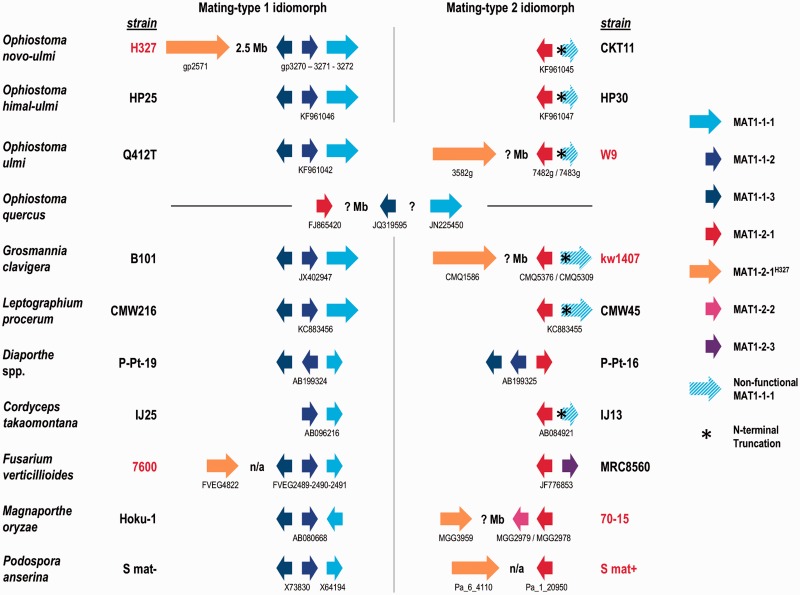
Mating-type idiomorph in the *O. novo-ulmi* H327 genome compared with other Sordariomycetes. For each fungus, an example of a mating-type 1 strain (to the left) and a mating-type 2 strain (to the right) are given. Where possible, fully sequenced genomes were used as the examples and they are indicated by red strain names. For simplicity, sizes are roughly relative to final protein sizes (small ≤ 500 aa, medium = 500–1,000 aa, large ≥ 1,000 aa), not gene lengths, and variable intergenic spaces are ignored. Individual gene names are given for those from completely sequenced genomes (according to the respective projects’ nomenclatures—see publications or genome browser websites), otherwise GenBank nucleotide accession numbers are given. The whole locus (between conserved regions upstream and downstream of the MAT genes) was sequenced in all cases except *O. quercus* strains (hence it is not known whether MAT1-1-2 is truly absent, nor how much distance is between the MAT1-1 genes and the MAT1-2-1 gene) where just pieces of individual genes were amplified with specific primers. Note that all sequenced strains of *O. quercus* (of either type) so far have the same topology and the example given is for strain CMW2521. For three whole genome strains (W9, kw1407, and 70-15), complete chromosomes have not been assembled, therefore it is unknown whether the MAT1-2-1^H327^ genes are truly on the same chromosomes as the other MAT genes (an unknown distance apart, indicated by “? Mb”). However, two of the whole genome strains (7600 and S mat + ) do have complete chromosomes available and the MAT1-2-1^H327^ genes are on separate chromosomes (indicated by a nonapplicable [n/a] distance apart). Note that only the *O. novo-ulmi* subspecies *novo-ulmi* strain topologies are shown, but that the strains of subspecies *americana* sequenced in this study (VA [type 1 = KF961043] and MH75 [type 2 = KF961044]) are identical, with the exception of the distant MAT1-2-1^H327^ gene of course whose status is unknown in the others as only the MAT locus was sequenced.

Sexual reproduction in ascomycete fungi is controlled generally by two mating types (idiomorphs), labeled “A” and “B” or “1” and “2,” akin to the “a” and “α” mating types in *Saccharomyces*, controlled by the *MAT* genes (Debuchy and Turgeon 2006; Haber 2012). Within the *Ophiostoma* spp., some are homothallic, containing both mating-types (1 and 2) within the same strain and therefore capable of self-fertilization (selfing). The DED pathogens *O. ulmi/novo-ulmi/himal-ulmi*, along with the phylogenetically closely related sapstain fungus *Ophiostoma quercus*, however, are heterothallic, with each strain containing only one idiomorph and requiring strains of opposing type for sexual reproduction (Paoletti et al. 2005; Wilken et al. 2012). The *O. novo-ulmi* strain H327 is of mating type 1 and its three *MAT* genes (*MAT1-1-1*, *MAT1-1-2,* and *MAT1-1-3*) had already been sequenced and characterized (FJ858801; Jacobi et al. 2010). Examination of the genome assembly confirmed their location in a cluster on chr.II and a genome-to-genome alignment against the appropriate scaffold from *O. ulmi* W9 showed a large region (∼360 kb) around the MAT locus has very high identity (≥95% nt level) between the two (supplementary fig. S6, Supplementary Material online), consistent with the hypothesis of Paoletti et al. (2006) that the MAT1-1 locus was recently acquired by *O. novo-ulmi* from *O. ulmi*. Unexpectedly, a putative mating-type 2 gene was detected approximately 2.5 Mb upstream on the same chromosome (fig. 7). Various *MAT1-2-1* genes (from *Ophiostoma* spp. and others) we used in a targeted local BLASTp of the H327 genome detected homology in the C-terminal half of gp2571—best match was to *Fusarium* spp. (∼45% identity and ∼70% similarity). This gene is therefore quite divergent from the other “true” *MAT1-2-1* genes; however, Conserved Database Domain and Pfam searches confirmed significant hits to the MATA_HMG-box domain (e^−^^32^ to cd01389 and e^−^^21^ to PF00505, respectively), hallmark of the MAT1-2-1 proteins (Debuchy and Turgeon 2006; Martin et al. 2010). We propose to label this new, divergent mating-type 2 allele *MAT1-2-1^H^^327^*. Curiously, we have also been able to find homologs of this new allele in other sequenced Sordariomycete genomes (fig. 7): *O. ulmi* W9 (*3582_g*), *G. clavigera* kw1407 (*CMQ_1586*), *Podospora anserina* S mat + (*Pa_6_4110*), *Fusarium verticillioides* MRC8560 (*FVEG_4822*), and *Magnaporthe orzyae* 70-15 (*MGG_3959*). The existence of both potentially functional mating-types in the heterothallic H327 is interesting as a recent study on the heterothallic *O. quercus* also showed both standard type 1 and 2 idiomorphs could be amplified/sequenced in all strains tested (Wilken et al. 2012). Our own examination of the heterothallic *O. ulmi* W9 genome shows that it has an N-terminal-truncated nonfunctional copy of MAT1-1-1 in its MAT1-2 idiomorph, as do many other mating-type-2 Sordariomycetes (fig. 7). These data, along with mapping from Paoletti et al. (2006), suggest that the mating-type loci (especially MAT1-1) undergo frequent recombination and that some heterothallic members of the *Ophiostoma* genus may be homothallic, but we have yet to identify the proper culture conditions under which significant amounts of selfing will be observed. Alternatively, the mating type 2 in these fungi may be controlled by more than just the one classical *MAT1-2-1* gene, requiring (an) extra gene(s) such as in some other ascomycetes (Martin et al. 2010), and it would be these as-yet unidentified genes which are missing in the assumed heterothallic *Ophiostoma* spp. of mating type 1.

In order to quickly test the functionality of the divergent *MAT1-2-1^H^^327^* allele, we attempted to provoke selfing in strain H327. Nearly 40 years ago Brasier and Gibbs (1975) observed some unusual mating-type 2 strains of *O. novo-ulmi* that produced a small (∼2%), but easily observable frequency of selfing. However, we were not able to show formation of perithecia (sexual reproductive organs) from any H327 × H327 anastomoses (vegetative contacts) using a variety of techniques, nor with other strains (CKT11, VA, FG245, and W2) that were selfed. Perithecia only occurred in positive mating controls between strains carrying different *MAT1* alleles. Assuming the current known version of mating-type 2 being controlled by one gene only, this implies that the *MAT1-2-1^H^^327^* allele may represent a “degraded” version of the original *MAT1-2-1* alleles still functioning in true mating-type 2 strains. Finally, the above also implies that strain H327 might have been homothallic at one time and subsequently lost this ability as its *MAT1-2-1* idiomorph degraded, forcing it into its current heterothallic sexual state.

Another mechanism, and gene set, involved in controlling anastomoses is the vegetative (or heterokaryon) incompatibility (VI) complex (*vic* or *het* loci). Fungi have the unusual capacity of undergoing vegetative cell fusion, creating heterokaryotic cells, and a large number of *het* loci establish whether the heterokaryons will remain viable (between genetically similar individuals) or be inhibited/destroyed (between genetically distinct individuals) by programmed cell death (Saupe 2000; Saupe et al. 2000). The genome of H327 has 35 genes/loci potentially involved in the VI system distributed throughout all eight chromosomes (supplementary fig. S7, Supplementary Material online). There are multiple copies of the typical *het-C/D/E/6* genes, along with single copies of homologs of *un-24* and *vib-1* from *Neurospora* which contain ribonucleotide reductase and transcriptional regulator domains, respectively, and play other cellular roles in addition to being involved in VI (Saupe 2000; Xiang and Glass 2004; Dementhon et al. 2006). There are also two copies of *het-R*, one of the prion-encoding *het-S* (fairly divergent) from *Podospora* (Chevanne et al. 2009; Saupe 2011) and a collection of nine genes encoding proteins with Het domains (Het-X), but which are too divergent to assign to any of the other known types. Aside from the above genes directly implicated in controlling/participating in VI, there are six genes in H327 that belong to the *mod-A/D/E* family of genes which can suppress VI if mutated (Saupe 2000).

### Pathogenicity Genes/Pathways

#### Overview

A major, long-term goal of sequencing and analyzing the H327 genome is to identify and understand the mechanisms controlling phytopathogenicity. The SMURF system (Khaldi et al. 2010) identified seven potential clusters, containing 2–18 genes each (table 3, supplementary fig. S8, Supplementary Material online), of fungal secondary metabolites that may be involved. The PHI-base system (Winnenberg et al. 2008) identified 1,731 genes potentially involved in pathogen–host interactions (table 3), including 850 genes that are known to reduce or abolish pathogenicity if mutated. These gene lists are obviously prime targets for future experimentation (e.g., knockouts, RNA silencing, transcription studies, etc.) and we will present below examples of proteins and pathways that we believe are some of the key phytopathogenicity components in *O. novo-ulmi*. Of note, the well-known cerato-ulmin hydrophobin (Temple et al. 1997; Temple and Horgen 2000) was identified on chr.III, but we concentrate below on more novel insights from so-far less-studied genes/pathways.

#### Peptidases and CAZymes against Plant Defenses

As mentioned above, six exported peptidases in H327 are only active on terminal prolines and seven others are active on small oligopeptides. This latter group may simply indicate peptidases which aid in the final nutritional degradation of initially larger proteins, or it may indicate a particular phytopathogenic response to destroy/modify elm antifungal peptides. It is well known that plants produce a wide variety of antimicrobial peptides (e.g., defensins, thionins, etc.) and much work is currently being done in vitro and in planta (transgenic plants) to test their efficacy against phytopathogens (Odintsova and Egorov 2012; Stotz et al. 2013). Some antimicrobial peptides have already been shown to be active in vitro against *O. ulmi* (Jacobi et al. 2000) and transgenic American elms expressing a synthetic peptide have shown reduced DED symptoms when infected with *O. novo-ulmi* (Newhouse et al. 2007). Aoun et al. (2010) also showed that one of the most highly upregulated genes in callus cultures of American elm during *O. novo-ulmi* infection was a homolog of (pseudo-)heveins, small peptides with antifungal activity by binding to chitin (Odintsova and Egorov 2012; Stotz et al. 2013). One can suspect that some of the H327 oligopeptidases may be involved in combating this host defense.

The group of terminal-proline peptidases is interesting because biochemical data from over 40 years ago showed a particular relationship between proline and DED. In fact, proline was present in considerable quantities in the sap of DED-resistant Ulmaceae versus being in trace amounts in susceptible species (Singh and Smalley 1969a); additionally, these authors found large increases of proline in sap after infection by *O. ulmi* (Singh and Smalley 1969b). There is some evidence from a decade later that proline can control the dimorphism—the transition between yeast and mycelial states—of *Ophiostoma* spp. (Kulkarni and Nickerson 1981), but our recent data suggest that the response is variable inter- and intraspecifically for the DED fungi (Naruzawa and Bernier 2014). However, it is now known that proline/hydroxyproline-rich glycoproteins (P/HRGPs) play major roles in plant defense against mechanical and phytopathogen attacks (Sommer-Knudsen et al. 1998; Deepak et al. 2010). The P/HRGPs, which include the extensins and lectins, act in three aspects of defense: 1) Chemical and structural remodeling of the plant cell wall to prevent invasion into tissues, 2) inhibition of phytopathogen-derived plant-cell-wall-degrading enzymes (i.e., CAZymes), and 3) antimicrobial killing/inhibition of phytopathogens by direct interaction (e.g., through chitin-binding activity). It therefore makes much sense for phytopathogenic *Ophiostoma* spp. to have a complement of exported peptidases that may be specifically targeted to host defense P/HRGPs. This scenario also ties back to H327’s CAZyme complement in two ways: First, given the capacity of P/HRGPs to inhibit certain CAZymes, *Ophiostoma* spp. are under selective pressure to modify their CAZymes to avoid detection/inactivation; second, as P/HRGPs are glycoproteins, there is also selective pressure to adapt CAZymes to specifically target these molecules directly for degradation, allowing a two-pronged attack against P/HRGPs with the above-mentioned peptidase activities.

#### Cytochromes (and Associated Genes) to Detoxify Plant Defenses and Synthesize Toxins

As previously mentioned, 48 CYP450s were identified in the H327 genome and the majority is potentially involved in phytopathogenesis or toxin production (supplementary table S8, Supplementary Material online). Nine are apparent pisatin demethylases, which detoxify the phytoalexin ( = plant defense; Grayer and Kokubun 2001) pisatin. Although this enzymatic capacity is apparently common in fungi (Delserone et al. 1999), these CYP450s may target other members of the broader group of defense (iso)flavonoids (Treutter 2006) to which pisatin belongs. In a similar fashion, seven other CYP450s appear to be involved in detoxifying as-yet undetermined benzene/phenolic compounds—an additional larger category of general phytoalexins, within which flavonoids themselves are found (Dixon et al. 2002; Daayf et al. 2012). Biochemical studies and EST analysis in vitro and in planta have shown that some of the major phytoalexins produced by the American elm in response to *O. novo-ulmi* infection are (iso)flavonoids, phenolics, and mansonones (Duchesne et al. 1985, 1994; Aoun et al. 2010). In a comparative study of mycelial growth kinetics, *O. novo-ulmi* was found to be more tolerant than most of the 17 filamentous fungi tested on solid media containing mansonone E (Proctor et al. 1994). Mansonones are sesquiterpenoid quinones and therefore it is highly likely that they would also be preferred targets for some of the above-mentioned CYP450s and accessory genes.

Nine CYP450s appear to be putatively involved in mycotoxin synthesis—three for trichothecenes and six for ST. The three former CYP450s are joined by two non-CYP genes, a putative trichothecene acetyltransferase (*g6369*) and efflux pump (*g7758*); however, they are not in a cluster as is generally the case (Merhej et al. 2011). Additionally, although the genes present would seem to catalyze the final steps in the production of the simplest trichothecene toxin trichodermin (McCormick et al. 2011), a homolog of the Tri5 trichodiene synthase which starts the pathway cannot be found in the H327 genome. It is therefore unknown to what extent these genes may be functional/implicated in true toxin production. The situation with the ST-associated CYP450s appears to be somewhat different, however, with at least one (CYP5442A1) being present in a cluster on chr.VII (identified by SMURF; supplementary fig. S8, Supplementary Material online) with an efflux transporter and two putative ST 8-*O*-methyltransferases (StcP, ST → 8-*O*-methylST [OMST]). Importantly, this cluster is built upon a polyketide synthase (possibly StcA)—ST and aflatoxin are both polyketide-derived products, with ST being the penultimate precursor of aflatoxin B1/G1 (ST → OMST → aflatoxin; Yu et al. 2004; Yu 2012). This marks the largest degree of clustering, however, as the remainder of the genes are scattered throughout the H327 genome. Besides six OMST oxidoreductase CYP450s (OMST → aflatoxin) and the two methyltransferases above, there are nine genes annotated as putative ST biosynthesis monooxygenases (StcW + undefined) that are probably involved in some of the ten steps from the polyketide precursor to ST; and two additional genes immediately identifiable in the pathway: CYP65BB4 (StcF) and *g5028* (StcE). Finally, there are two genes (*g0943* and *g3733*) putatively identified as encoding aflatoxin efflux transporters. Therefore, although it remains to be shown biochemically, it appears probable that *O. novo-ulmi* H327 can produce ST and aflatoxin—such a capacity, especially for ST, appears to be somewhat common within members of the Pezizomycotina (Rank et al. 2011) and its presence in multiple phytopathogens implies a conferred advantage when attacking plant hosts.

#### Specific Interactions with Plant Defense Terpenes

As mentioned previously, one CYP450 (*g7466*, CYP52P6; supplementary table S8, Supplementary Material online) seemed to be specifically involved in modifying plant terpene defenses. This CYP450 was pulled out of the genome by local BLASTp using a collection of vector beetle CYP450s (*Dendroctonus* spp.; Cano-Ramírez et al. 2013) that were implicated in transformations of α-pinene, possibly to verbenol. We originally interested ourselves in this transformation as the end-product verbenol is an aggregation pheromone for bark beetles (Blomquist et al. 2010) which increases the intensity of the infection foyer and thereby increases dissemination efficiency of vector-associated phytopathogenic fungi such as *Grosmannia* and *Ophiostoma*. Although H327’s CYP52P6 had closest matches (61–69% similarity) to unknown-specificity fungal CYP450s, it also showed a moderate match (54% aa similarity) to a *Meyerozyma* CYP450 (AFN08702.1) recently directly implicated in the α-pinene → *cis*-verbanol transformation, although details of this study are as of yet unpublished. Given that aa similarities between the CYP52P6, *Meyerozyma,* and the various bark beetle CYP450s are only in the range of 40–55%, there is potential for differing substrate specificities. It is also well known that trees, including members of the Ulmaceae, produce a large suite of related terpenes (including the highly abundant limonene) and that infecting fungi may be responsible for stimulating the tree’s overproduction of these compounds and/or they may directly degrade/transform many of them (Duetz et al. 2003; McLeod et al. 2005; Rottava et al. 2010).

In order to investigate the potential response(s) of *O. novo-ulmi* H327 to terpenes, two complementary approaches were undertaken. Liquid (yeast form) and solid (mycelial form) cultures of H327 were grown in the presence of α-pinene, β-pinene, and limonene to observe their effect on base growth-rates. Second, liquid and solid cultures were exposed to these same terpenes and time course experiments were then conducted in order to quantify *g7466* (CYP52P6) expression profiles through qPCR. In the first case, yeast growth kinetics were negatively impacted by the presence of terpenes in liquid media, at all concentrations tested. At lower concentrations (0.05% and 0.1% vol/vol), α-pinene and β-pinene delayed the onset of the exponential growth phase by approximately 50 h, whereas cultures exposed to limonene took approximately 100 h to enter exponential growth phase. Growth of yeast cell samples exposed to 0.5% limonene or to 1.0% α-pinene or β-pinene was completely inhibited during the 14 days of incubation. In contrast, mycelium of H327 growing on solid media showed substantial resistance to α-pinene or β-pinene up to concentrations of 1.0% vol/vol, similar to the terpene resistance (and utilization) encountered in the other phytopathogen *G. clavigera* (DiGuistini et al. 2011) contrasted with the fungi-static effects seen in the saprophyte *O. piceae* (Haridas et al. 2013). Interestingly, limonene had a stimulatory effect—mycelial growth rate of H327 increased nearly 2-fold at 0.05–0.5% vol/vol.

Limonene, one of the most abundant and effective defense monoterpenes (Langenheim 1994; Wise and Croteau 1999; Boone et al. 2011; Schiebe et al. 2012), could therefore stimulate the more tissue-invasive mycelial state in *O. novo-ulmi* as well as promote mycelial growth in elm bark beetle galleries during the fungus saprophytic phase. Products of limonene degradation are numerous (Duetz et al. 2003), so it is currently unknown whether H327’s response would also affect beetle pheromone signaling (e.g., terpineol end-product [Lacey et al. 2008]) or simply detoxify its local environment. Interestingly, Hubbes (1975) found that both limonene and terpineol induced the formation of asexual reproductive synnemata—the structures containing the spores which “paint” *O. novo-ulmi*’s insect vector. Finally, regardless of terpene type or culture state (solid or liquid), qPCR-measured expression patterns of CYP52P6 were unfortunately inconclusive. More in-depth biochemical studies (specific degradation products) and rigorous expression profiling (RNA-seq) are planned in order to clarify the exact function of this, or other, CYP450s involved in terpene interactions.

## Conclusion

Our full biological annotation of the *O. novo-ulmi* H327 nuclear genome should allow insights into main mechanisms controlling growth, pathogenicity, and relationships with other members of the pathosystem. This work has identified prime targets for future genetic manipulations toward the long-term goal of understanding the mechanisms controlling phytopathogenicity and population spread of this important fungal species (Bernier et al. 2014).

## Supplementary Material

Supplementary figures S1–S8, tables S1–S8, and datafiles S1–S3 are available at *Genome Biology and Evolution* online (http://www.gbe.oxfordjournals.org/).

Supplementary Data

## References

[evu281-B1] Amselem J (2011). Genomic analysis of the necrotrophic fungal pathogens *Sclerotinia sclerotiorum* and *Botrytis cinerea*. PLoS Genet..

[evu281-B2] Aoun M, Jacobi V, Boyle B, Bernier L (2010). Identification and monitoring of *Ulmus americana* transcripts during *in vitro* interactions with the Dutch elm disease pathogen *Ophiostoma novo-ulmi*. Physiol Mol Plant Pathol..

[evu281-B3] Aspeborg H, Coutinho PM, Wang Y, Brumer H, Henrissat B (2012). Evolution, substrate specificity and subfamily classification of glycoside hydrolase family 5 (GH5). BMC Evol Biol..

[evu281-B4] Bernier L, Seifert KA, Wingfield MJ (2013). Towards ophiostomatoid genomics.

[evu281-B5] Bernier L (2014). Genomics of the Dutch elm disease pathosystem: are we there yet? iForest. Advance Access published August 7, 2014.

[evu281-B6] Bernier L, Hubbes M (1990). Mutations in *Ophiostoma ulmi* induced by *N*-methyl-*N'*-nitro-*N*-nitrosoguanidine. Can J Bot..

[evu281-B7] Blomquist GJ (2010). Pheromone production in bark beetles. Insect Biochem Mol Biol..

[evu281-B8] Boone CK, Aukema BH, Bohlmann J, Carroll AL, Raffa KF (2011). Efficacy of tree defense physiology varies with bark beetle population density: a basis for positive feedback in eruptive species. Can J For Res..

[evu281-B9] Bouvet GF, Jacobi V, Bernier L (2007). Characterization of three DNA transposons in the Dutch elm disease fungi and evidence of repeat-induced point (RIP) mutations. Fungal Genet Biol..

[evu281-B10] Bouvet GF, Jacobi V, Plourde KV, Bernier L (2008). Stress-induced mobility of *OPHIO1* and *OPHIO2*, DNA transposons of the Dutch elm disease fungi. Fungal Genet Biol..

[evu281-B11] Brasier CM, Gibbs JN (1975). Highly fertile form of the aggressive strain of *Ceratocystis ulmi*. Nature.

[evu281-B12] Brasier CM, Kirk SA (2001). Designation of the EAN and NAN races of *Ophiostoma novo-ulmi* as subspecies. Mycol Res..

[evu281-B13] Brasier CM, Mehrotra MD (1995). *Ophiostoma himal-ulmi* sp. nov., a new species of Dutch elm disease fungus endemic to the Himalayas. Mycol Res..

[evu281-B14] Cano-Ramírez C (2013). Isolation and expression of cytochrome P450 genes in the antennae and gut of pine beetle *Dendroctonus rhizophagus* (Curculionidae: Scolytinae) following exposure to host monoterpenes. Gene.

[evu281-B15] Cantarel BL (2009). The Carbohydrate-Active EnZymes database (CAZy): an expert resource for Glycogenomics. Nucleic Acids Res..

[evu281-B16] Carver T, Harris SR, Berriman M, Parkhill J, McQuillan JA (2012). Artemis: an integrated platform for visualization and analysis of high-throughput sequence-based experimental data. Bioinformatics.

[evu281-B17] Chevanne D (2009). Identification of the *het-r* vegetative incompatibility gene of *Podospora anserina* as a member of the fast evolving *HNWD* gene family. Curr Genet..

[evu281-B18] Clutterbuck AJ (2011). Genomic evidence of repeat-induced point mutation (RIP) in filamentous ascomycetes. Fungal Genet Biol..

[evu281-B19] Cohn M, Liti G, Barton DBH (2005). Telomeres in fungi. Top Curr Genet..

[evu281-B20] Cuomo CA (2014). Genome sequence of the pathogenic fungus *Sporothrix schenckii* (ATCC 58251). Genome Announc..

[evu281-B21] Daayf F, Cheynier V, Sarni-Manchado P, Quideau S (2012). Phenolic compounds in plant defense and pathogen counter-defense mechanisms. Recent advances in polyphenol research.

[evu281-B22] Dean RA (2005). The genome sequence of the rice blast fungus *Magnaporthe grisea*. Nature.

[evu281-B23] de Beer ZW, Duong TA, Barnes I, Wingfield DB, Wingfield MJ (2014). Redefining *Ceratocystis* and allied genera. Stud Mycol..

[evu281-B24] Debuchy R, Turgeon BG, Kües U, Fischer R (2006). Mating-type structure, evolution, and function in Euascomycetes. The Mycota I: growth, differentiation and sexuality.

[evu281-B25] Deepak S (2010). Hydroxyproline-rich glycoproteins and plant defence. J Phytopathol..

[evu281-B26] Delserone LM, McCluskey K, Matthews DE, Vanetten HD (1999). Pisatin demethylation by fungal pathogens and nonpathogens of pea: association with pisatin tolerance and virulence. Physiol Mol Plant Pathol..

[evu281-B27] Dementhon K, Iyer G, Glass NL (2006). VIB-1 is required for expression of genes necessary for programmed cell death in *Neurospora crassa*. Eukaryot Cell..

[evu281-B28] de Souza FS, Franchini LF, Rubinstein M (2013). Exaptation of transposable elements into novel *cis*-regulatory elements: is the evidence always strong?. Mol Biol Evol..

[evu281-B29] Dewar K, Bernier L (1995). Inheritance of chromosome-length polymorphisms in *Ophiostoma ulmi* (*sensu lato*). Curr Genet..

[evu281-B30] Dewar K, Bousquet J, Dufour J, Bernier L (1997). A meiotically reproducible chromosome length polymorphism in the ascomycete fungus *Ophiostoma ulmi* (*sensu lato*). Mol Gen Genet..

[evu281-B31] de Wit PJGM (2012). The genomes of the fungal plant pathogens *Cladosporium fulvum* and *Dothistroma septosporum* reveal adaptation to different hosts and lifestyles but also signatures of common ancestry. PLoS Genet..

[evu281-B32] DiGuistini S (2009). *De novo* genome sequence assembly of a filamentous fungus using Sanger, 454 and Illumina sequence data. Genome Biol..

[evu281-B33] DiGuistini S (2011). Genome and transcriptome analyses of the mountain pine beetle-fungal symbiont *Grosmannia clavigera*, a lodgepole pine pathogen. Proc Natl Acad Sci U S A..

[evu281-B34] Dixon RA (2002). The phenylpropanoid pathway and plant defence—a genomics perspective. Mol Plant Pathol..

[evu281-B35] Duchesne LC, Jeng RS, Hubbes M (1985). Accumulation of phytoalexins in *Ulmus americana* in response to infection by a nonaggressive and an aggressive strain of *Ophiostoma ulmi*. Can J Bot..

[evu281-B36] Duchesne LC, Jeng RS, Hubbes M, Sticklen MB (1994). Accumulation of mansonones E and F in elm callus cultures inoculated with *Ophiostoma ulmi*. Can J Plant Pathol..

[evu281-B37] Duetz WA, Bouwmeester H, van Beilen JB, Witholt B (2003). Biotransformation of limonene by bacteria, fungi, yeasts, and plants. Appl Microbiol Biotechnol..

[evu281-B38] Ellison CE, Bachtrog D (2013). Dosage compensation via transposable element mediated rewiring of a regulatory network. Science.

[evu281-B39] Espagne E (2008). The genome sequence of the model ascomycete fungus *Podospora anserina*. Genome Biol..

[evu281-B40] Et-Touil A, Brasier CM, Bernier L (1999). Localization of a pathogenicity gene in *Ophiostoma novo-ulmi* and evidence that it may be introgressed from *O. ulmi*. Mol Plant Microbe Interact..

[evu281-B41] Forgetta V (2013). Sequencing of the Dutch elm disease fungus genome using the Roche/454 GS-FLX Titanium system in a comparison of multiple genomics core facilities. J Biomol Tech..

[evu281-B42] Galagan JE (2003). The genome sequence of the filamentous fungus *Neurospora crassa*. Nature.

[evu281-B43] Grayer RJ, Kokubun T (2001). Plant-fungal interactions: the search for phytoalexins and other antifungal compounds from higher plants. Phytochemistry.

[evu281-B44] Haber JE (2012). Mating-type genes and MAT switching in *Saccharomyces cerevisiae*. Genetics.

[evu281-B45] Hane JK, Oliver RP (2008). RIPCAL: a tool for alignment-based analysis of repeat-induced point mutations in fungal genomic sequences. BMC Bioinformatics.

[evu281-B46] Haridas S (2013). The genome and transcriptome of the pine saprophyte *Ophiostoma piceae*, and a comparison with the bark beetle-associated pine pathogen *Grosmannia clavigera*. BMC Genomics.

[evu281-B47] Harris JL, Taber WA (1970). Influence of certain nutrients and light on growth and morphogenesis of the synnema of *Ceratocystis ulmi*. Mycologia.

[evu281-B48] Hartley C, Davidson RW, Crandall BS (1961).

[evu281-B49] Hintz W (2011). Functional categorization of unique expressed sequence tags obtained from the yeast-like growth phase of the elm pathogen *Ophiostoma novo-ulmi*. BMC Genomics.

[evu281-B50] Holmes FW, Heybroek HM (1990). Dutch elm disease—the early papers.

[evu281-B51] Hubbes M (1975). Terpenes and unsaturated fatty acids trigger coremia formation by *Ceratocystis ulmi*. Eur J For Pathol..

[evu281-B52] Jacobi V, Dufour J, Bouvet GF, Aoun M, Bernier L (2010). Identification of transcripts up-regulated in asexual and sexual fruiting bodies of the Dutch elm disease pathogen *Ophiostoma novo-ulmi*. Can J Microbiol..

[evu281-B53] Jacobi V, Plourde A, Charest PJ, Hamelin RC (2000). *In vitro* toxicity of natural and designed peptides to tree pathogens and pollen. Can J Bot..

[evu281-B54] Jurka J (2005). Repbase Update, a database of eukaryotic repetitive elements. Cytogent Genome Res..

[evu281-B55] Kelly DE, Krasevec N, Mullins J, Nelson DR (2009). The CYPome (Cytochrome P450 complement) of *Aspergillus nidulans*. Fungal Genet Biol..

[evu281-B56] Khaldi N (2010). SMURF: genomic mapping of fungal secondary metabolite clusters. Fungal Genet Biol..

[evu281-B57] Khoshraftar S (2013). Sequencing and annotation of the *Ophiostoma ulmi* genome. BMC Genomics.

[evu281-B58] Kim D (2013). TopHat2: accurate alignment of transcriptomes in the presence of insertions, deletions and gene fusions. Genome Biol..

[evu281-B59] Krumsiek J, Arnold R, Rattei T (2007). Gepard: a rapid and sensitive tool for creating dotplots on genome scale. Bioinformatics.

[evu281-B60] Kulkarni RK, Nickerson KW (1981). Nutritional control of dimorphism in *Ceratocystis ulmi*. Exp Mycol..

[evu281-B61] Lacey ES, Moreira JA, Millar JG, Hanks LM (2008). A male-produced aggregation pheromone blend consisting of alkanediols, terpenoids, and an aromatic alcohol from the Cerambycid beetle *Megacyllene caryae*. J Chem Ecol..

[evu281-B62] Lagesen K (2007). RNAmmer: consistent and rapid annotation of ribosomal RNA genes. Nucleic Acids Res..

[evu281-B63] Lah L, Haridas S, Bohlmann J, Breuil C (2013). The cytochromes P450 of *Grosmannia clavigera*: genome organization, phylogeny, and expression in response to pine host chemicals. Fungal Genet Biol..

[evu281-B64] Langenheim JH (1994). Higher plant terpenoids: a phytocentric overview of their ecological roles. J Chem Ecol..

[evu281-B65] Lombard V, Golaconda Ramulu H, Drula E, Coutinho PM, Henrissat B (2014). The carbohydrate-active enzymes database (CAZy) in 2013. Nucleic Acids Res..

[evu281-B66] Martin T (2010). Tracing the origin of the fungal α1 domain places its ancestor in the HMG-box superfamily: implication for fungal mating-type evolution. PLoS One.

[evu281-B67] McCormick SP, Stanley AM, Stover NA, Alexander NJ (2011). Trichothecenes: from simple to complex mycotoxins. Toxins.

[evu281-B68] McLeod G (2005). The pathogen causing Dutch elm disease makes host trees attract insect vectors. Proc R Soc Lond B Biol Sci..

[evu281-B69] Merhej J, Richard-Forget F, Barreau C (2011). Regulation of trichothecene biosynthesis in *Fusarium*: recent advances and new insights. Appl Microbiol Biotechnol..

[evu281-B70] Monod M (2002). Secreted protease from pathogenic fungi. Int J Med Microbiol..

[evu281-B71] Moriya Y, Itoh M, Okuda S, Yoshizawa A, Kanehisa M (2007). KAAS: an automatic genome annotation and pathway reconstruction server. Nucleic Acids Res..

[evu281-B72] Naruzawa ES, Bernier L (2014). Control of yeast-mycelium dimorphism *in vitro* in Dutch elm disease fungi by manipulation of specific external stimuli. Fungal Biol..

[evu281-B73] Nelson DR, Phillips IR, Shephard EA (2006). Cytochrome P450 nomenclature, 2004. Methods in molecular biology, Vol. 320: Cytochrome P450 protocols.

[evu281-B74] Nelson DR (2009). The cytochrome P450 homepage. Hum Genomics..

[evu281-B75] Newhouse AE, Schrodt F, Liang H, Maynard CA, Powell WA (2007). Transgenic American elm shows reduced Dutch elm disease symptoms and normal mycorrhizal colonization. Plant Cell Rep..

[evu281-B76] Odintsova T, Egorov T, Irving HR, Gehring C (2012). Plant antimicrobial peptides. Plant signaling peptides. Signaling and communications in plants.

[evu281-B77] Olivieri F, Zanetti ME, Oliva CR, Covarrubias AA, Casalongué CA (2002). Characterization of an extracellular serine protease of *Fusarium eumartii* and its action on pathogenesis related proteins. Eur J Plant Pathol..

[evu281-B78] Paoletti M, Buck KW, Brasier CM (2005). Cloning and sequence analysis of the *MAT-B* (*MAT-2*) genes from the three Dutch elm disease pathogens, *Ophiostoma ulmi*, *O. novo-ulmi*, and O. himal-ulmi. Mycol Res..

[evu281-B79] Paoletti M, Buck KW, Brasier CM (2006). Selective acquisition of novel mating type and vegetative incompatibility genes via interspecies gene transfer in the globally invading eukaryote *Ophiostoma novo-ulmi*. Mol Ecol..

[evu281-B80] Petersen TN, Brunak S, von Heijne G, Nielsen H (2011). SignalP 4.0: discriminating signal peptides from transmembrane regions. Nat Methods..

[evu281-B81] Proctor RH, Guries RP, Smalley EB (1994). Lack of association between tolerance to the elm phytoalexin mansonone E and virulence in *Ophiostoma novo-ulmi*. Can J Bot..

[evu281-B82] Rank C (2011). Distribution of sterigmatocystin in filamentous fungi. Fungal Biol..

[evu281-B83] Rawlings ND, Barrett AJ, Bateman A (2012). MEROPS: the database of proteolytic enzymes, their substrates and inhibitors. Nucleic Acids Res..

[evu281-B84] Riordan JD, Dupuy AJ (2013). Domesticated transposable element gene products in human cancer. Mob Genet Elements..

[evu281-B85] Rottava I (2010). Microbial oxidation of (–)-α-pinene to verbenol production by newly isolated strains. Appl Biochem Biotechnol..

[evu281-B86] Saupe SJ (2000). Molecular genetics of heterokaryon incompatibility in filamentous ascomycetes. Microbiol Mol Biol Rev..

[evu281-B87] Saupe SJ (2011). The [Het-s] prion of *Podospora anserina* and its role in heterokaryon incompatibility. Semin Cell Dev Biol..

[evu281-B88] Saupe SJ, Clavé C, Bégueret J (2000). Vegetative incompatibility in filamentous fungi: *Podospora* and *Neurospora* provide some clues. Curr Opin Microbiol..

[evu281-B89] Schattner P, Brooks AN, Lowe TM (2005). The tRNAscan-SE, snoscan and snoGPS web servers for the detection of tRNAs and snoRNAs. Nucleic Acids Res..

[evu281-B90] Schiebe C (2012). Inducibility of chemical defenses in Norway spruce bark is correlated with unsuccessful mass attacks by the spruce bark beetle. Oecologia.

[evu281-B91] Singh D, Smalley EB (1969a). Nitrogenous and carbohydrate compounds in the xylem sap of Ulmaceae species varying in resistance to Dutch elm disease. Can J Bot..

[evu281-B92] Singh D, Smalley EB (1969b). Nitrogenous compounds in the xylem sap of American elms with Dutch elm disease. Can J Bot..

[evu281-B93] Sommer-Knudsen J, Bacic A, Clarke AE (1998). Hydroxyproline-rich plant glycoproteins. Phytochemistry.

[evu281-B94] Stam MR, Danchin EGJ, Rancurel C, Coutinho PM, Henrissat B (2006). Dividing the large glycoside hydrolase family 13 into subfamilies: towards improved functional annotations of α-amylase-related proteins. Protein Eng Des Sel..

[evu281-B95] Stanke M, Waack S (2003). Gene prediction with a hidden-Markov model and a new intron submodel. Bioinformatics.

[evu281-B96] Stotz HU, Waller F, Wang K, Hiemstra PS, Zaat SAJ (2013). Innate immunity in plants: the role of antimicrobial peptides. Antimicrobial peptides and innate immunity. Progress in Inflammation Research.

[evu281-B97] Temple B, Horgen PA (2000). Biological roles for cerato-ulmin, a hydrophobin secreted by the elm pathogens, *Ophiostoma ulmi* and O. novo-ulmi. Mycologia.

[evu281-B98] Temple B, Horgen PA, Bernier L, Hintz WE (1997). Cerato-ulmin, a hydrophobin secreted by the causal agents of Dutch elm disease, is a parasitic fitness factor. Fungal Genet Biol..

[evu281-B99] Ter-Hovhannisyan V, Lomsadze A, Chernoff Y, Borodovsky M (2008). Gene prediction in novel fungal genomes using an *ab initio* algorithm with unsupervised training. Genome Res..

[evu281-B100] Treutter D (2006). Significance of flavonoids in plant resistance: a review. Environ Chem Lett..

[evu281-B101] van der Nest MA (2014). Draft genome sequences of *Diplodia sapinea*, *Ceratocystis manginecans*, and *Ceratocystis moniliformis*. IMA Fungus.

[evu281-B102] Webber JF (1990). Relative effectiveness of *Scolytus scolytus*, *S. multistriatus*, and *S. kirschi* as vectors of Dutch elm disease. Eur J For Pathol..

[evu281-B103] Wicker T (2007). A unified classification system for eukaryotic transposable elements. Nat Rev Genet..

[evu281-B104] Wilken PM (2012). Both mating types in the heterothallic fungus *Ophiostoma quercus* contain *MAT1-1* and *MAT1-2* genes. Fungal Biol..

[evu281-B105] Wilken PM (2013). Draft nuclear genome sequence for the plant pathogen, *Ceratocystis fimbriata*. IMA Fungus.

[evu281-B106] Winnenberg R (2008). PHI-base update: additions to the pathogen-host interaction database. Nucleic Acids Res..

[evu281-B110] Wise ML, Croteau R, Cane DE (1999). Monoterpene biosynthesis. Comprehensive natural products chemistry: 2. Isoprenoids including carotenoids and steroids.

[evu281-B107] Xiang Q, Glass LN (2004). The control of mating type heterokaryon incompatibility by *vib-1*, a locus involved in *het-c* heterokaryon incompatibility in *Neurospora crassa*. Fungal Genet Biol..

[evu281-B108] Yu J (2012). Current understanding on aflatoxin biosynthesis and future perspective in reducing aflatoxin contamination. Toxins.

[evu281-B109] Yu J (2004). Clustered pathway genes in aflatoxin biosynthesis. Appl Environ Microbiol..

