# Supplementary material for: Functional Annotation of the Ophiostoma novo-ulmi Genome: Insights into the Phytopathogenicity of the Fungal Agent of Dutch Elm Disease
Source: Genome Biol Evol. 2014 Dec 24;7(2):410–30. doi: 10.1093/gbe/evu281 (PMC4350166; doi:10.1093/gbe/evu281)
Supplement: Supplementary Data [file supp_7_2_410__index.html]

Functional Annotation of the Ophiostoma novo-ulmi Genome: Insights into the Phytopathogenicity of the Fungal Agent of Dutch Elm Disease — Functional Annotation of the Ophiostoma novo-ulmi Genome: Insights into the Phytopathogenicity of the Fungal Agent of Dutch Elm Disease — Supplementary Data 

# Functional Annotation of the *Ophiostoma novo-ulmi* Genome: Insights into the Phytopathogenicity of the Fungal Agent of Dutch Elm Disease

## Supplementary Data

files

**Files in this Data Supplement:**

- Supplementary Data - zip file
