# Supplementary material for: Functional Annotation of the Ophiostoma novo-ulmi Genome: Insights into the Phytopathogenicity of the Fungal Agent of Dutch Elm Disease
Source: Genome Biol Evol. 2014 Dec 24;7(2):410–30. doi: 10.1093/gbe/evu281 (PMC4350166; doi:10.1093/gbe/evu281)

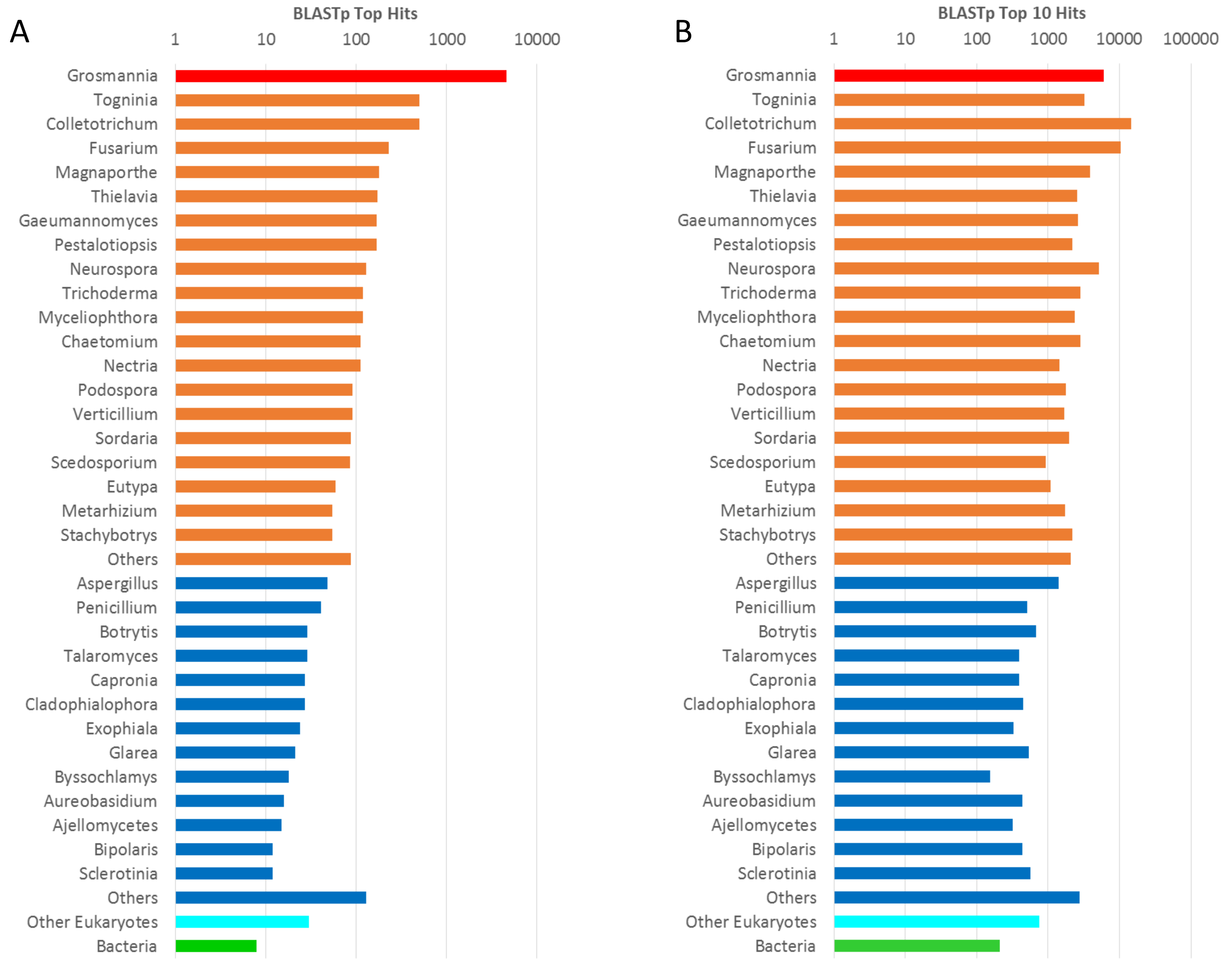

Fig. S1.—Taxonomic distributions of BLAST hits of *O. novo-ulmi* H327 proteins. (*A*) Number of top hits (1 per protein) for each organism. (*B*) Number of top 10 hits for each organism (same order as in *A*). Note color-coding matches Figure 2 and that BLASTs were conducted excluding the very close *Ophiostoma*/*Sporothrix* sister species (also as in Figure 2).

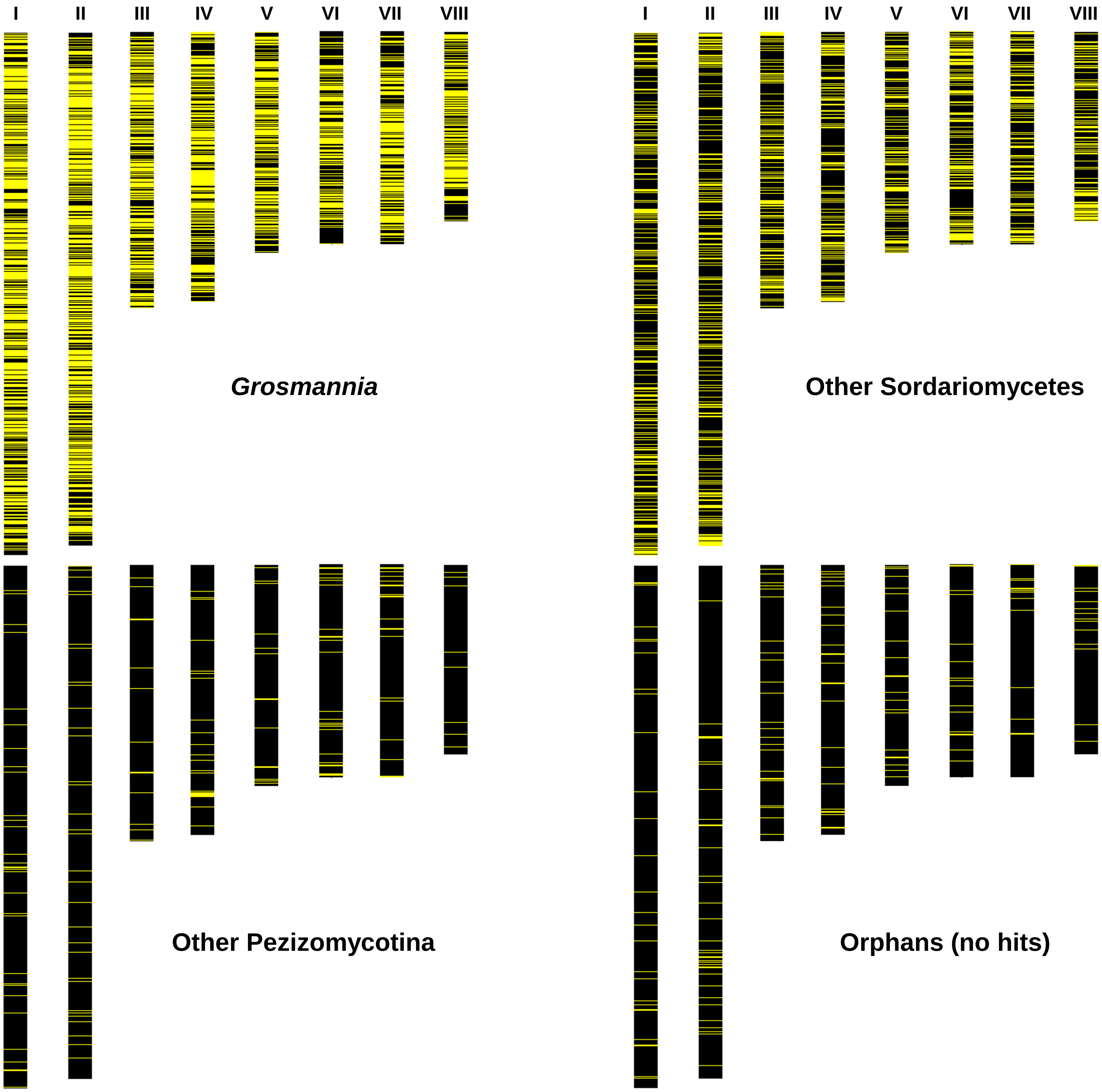

Fig. S2.—Quantity and distribution of homologs on the *O. novo-ulmi* H327 chromosomes. Layout of the figure is similar to Figure 2, except here yellow strips on black backgrounds represent top BLAST hits to the indicated taxa (or orphans).

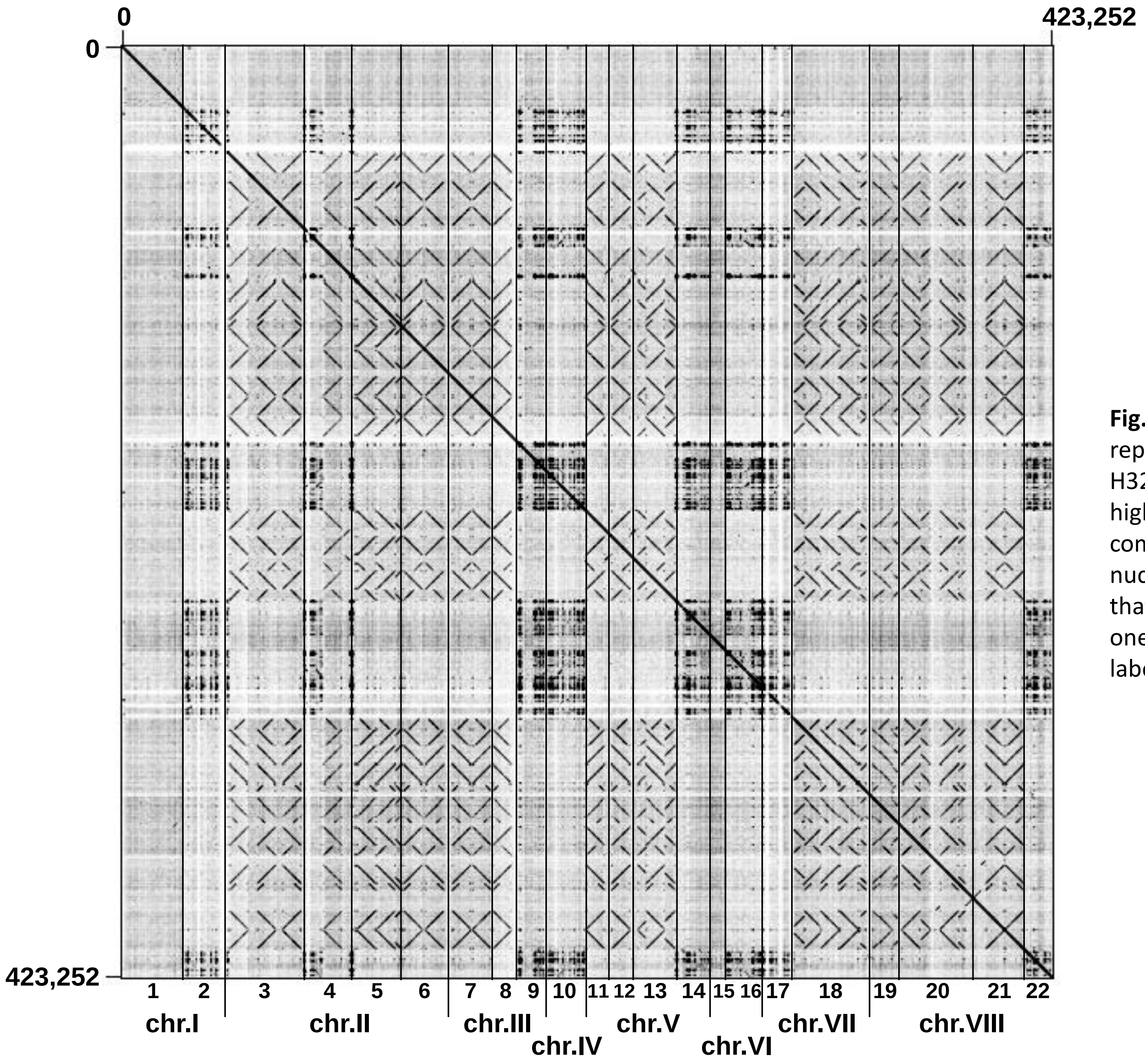

Fig. S3.—Dot-plot of the high-AT repeat regions of the *O. novo-ulmi* H327 chromosomes. The 22 regions highlighted in Figure 2 (by stars) are com-pared to themselves at the nucleotide level (using Gepard). Note that the x- and y-axes are mirrors of one another, even though only one is labeled.

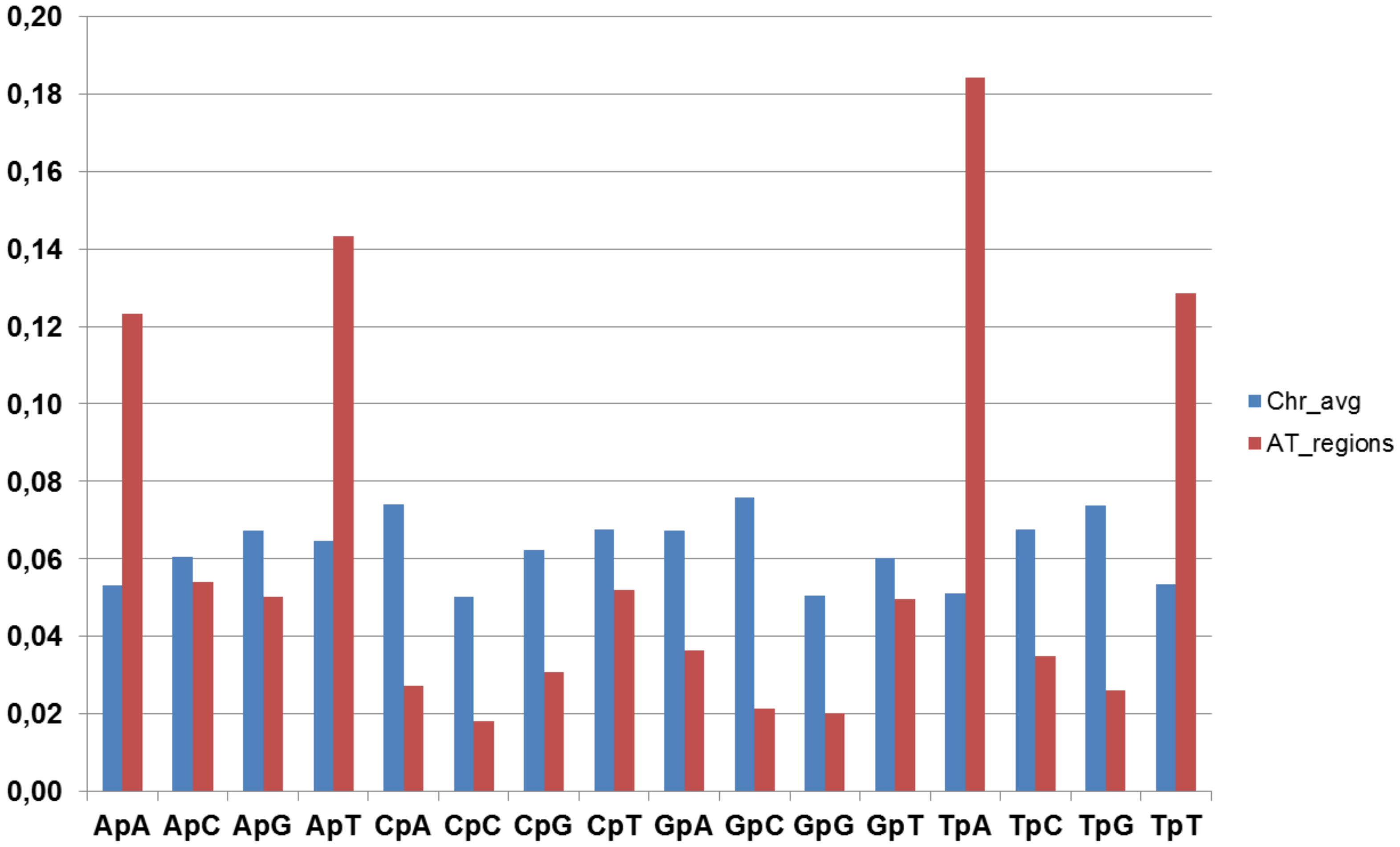

Fig. S4.—Dinucleotide frequencies of the high-AT repeat regions of the *O. novo-ulmi* H327 chromosomes. Frequencies were calculated with RIPCAL and are compared to the entire-genome average, indicating that the repeat regions show typical signs of RIP (ex: large depletion in CpA to TpA).

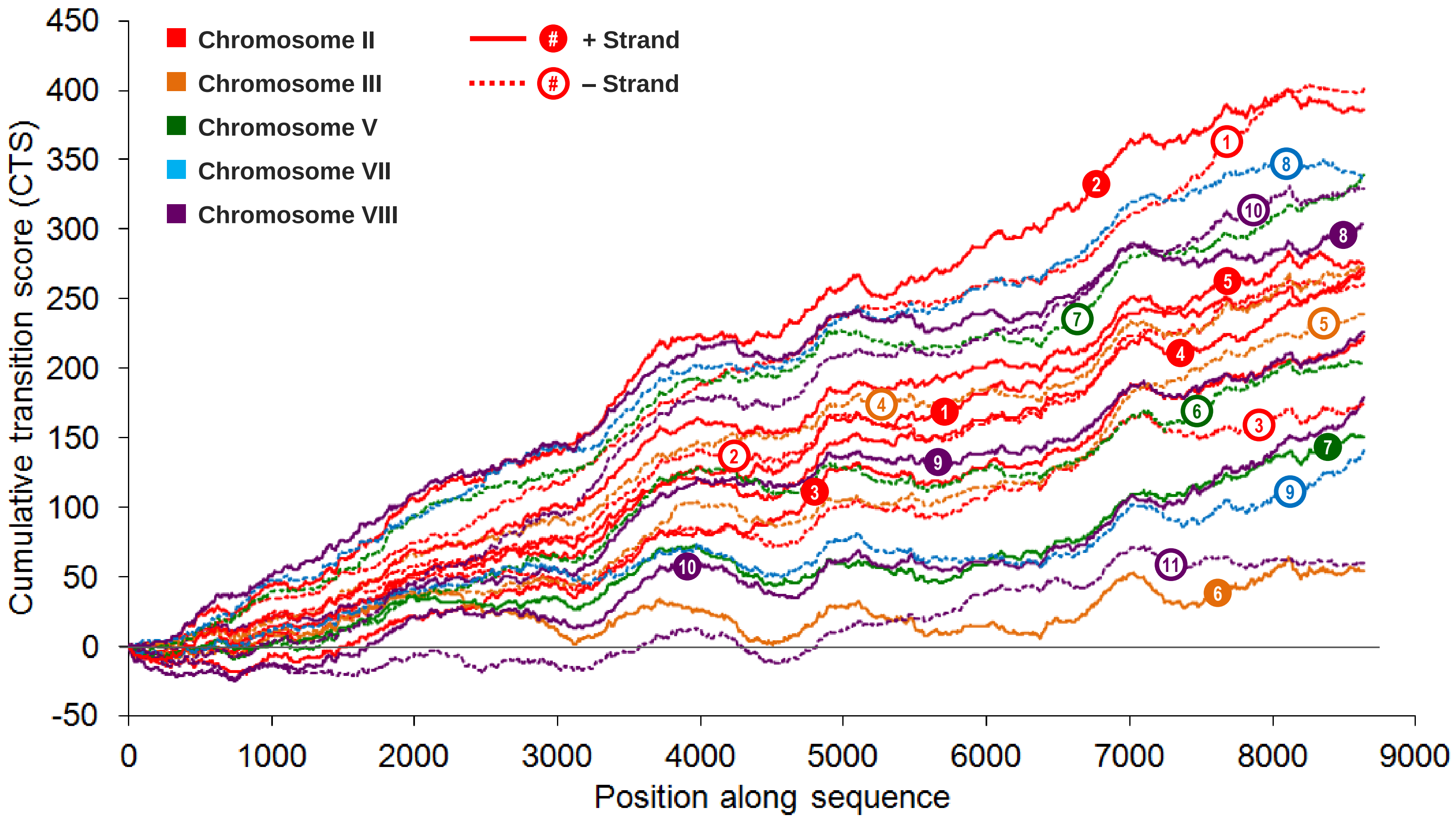

Fig. S5.—Cumulative transition scores (CTS) of newly annotated SWING retrotransposons in the *O. novo-ulmi* H327 genome. Most SWINGs show extensive RIP action by their large accumulation of transitions vis-à-vis the (internal) reference SWING (chrI:145-8,686). Positive-strand SWINGs: 1=chrII:1,076,851-1,085,392; 2=chrII:3,776,257-3,784,865; 3=chrII:5,942,755-5,951,297; 4=chrII:5,951,630-5,960,169; 5=chrII:5,983,162-5,991,702; 6=chrIII:1,230,867-1,239,406; 7=chrV:364,428-372,967; 8=chrVIII:490,874-499,407; 9=chrVIII:508,179-516,721; 10=chrVIII:1,525,943-1,534,481. Negative-strand SWINGs: 1=chrII:1,061,279-1,052,739; 2=chrII:1,073,868-1,065,325; 3=chrII:5,981,768-5,973,230; 4=chrIII:1,230,126-1,221,580; 5=chrIII:1,665,643-1,657,101; 6=chrV:618,548-610,007; 7=chrV:1,730,244-1,721,704; 8=chrVII:2,230,233-2,221,693; 9=chrVII:2,238,951-2,230,410; 10=chrVIII:1,525,566-1,517,025; 11=chrVIII:2,518,174-2,509,633. Note that one element (SWING-_chrVII:2,222,940-2,214,397) is very degraded and does not align well along the first ~1200 nt and hence has been excluded from this analysis/figure.

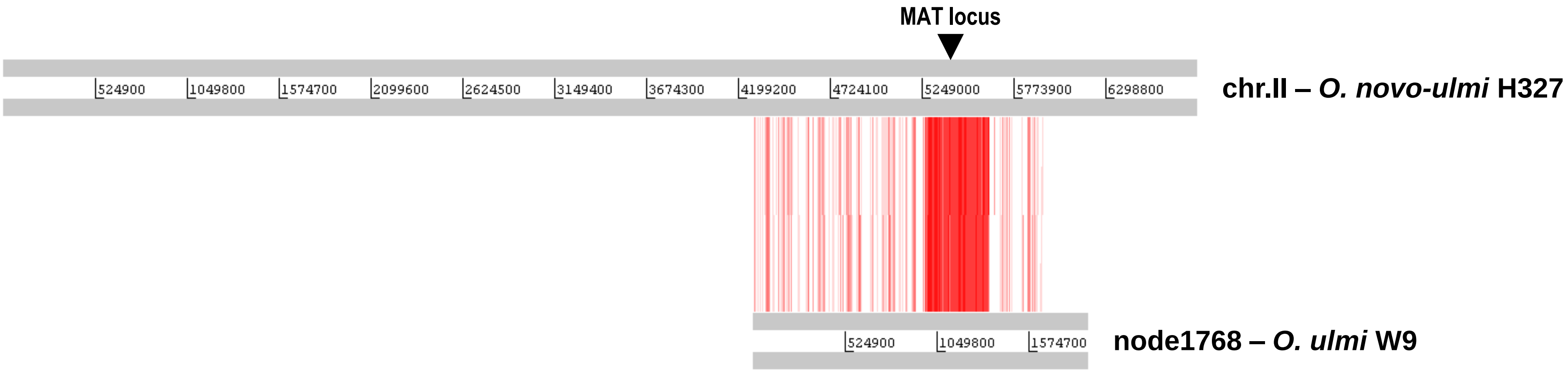

Fig. S6.—WebACT genome-to-genome comparison of *O. novo-ulmi* H327 vs. *O. ulmi* W9. H327 chromosome II is aligned to the W9 "node1768" scaffold in order to highlight the large (~360 kb) conserved region around the MAT locus between the two species. The vertical red bars between the sequences indicate ≥95% nucleotide identity and the denser they are (more closely packed together), the more contiguous the region of homology.

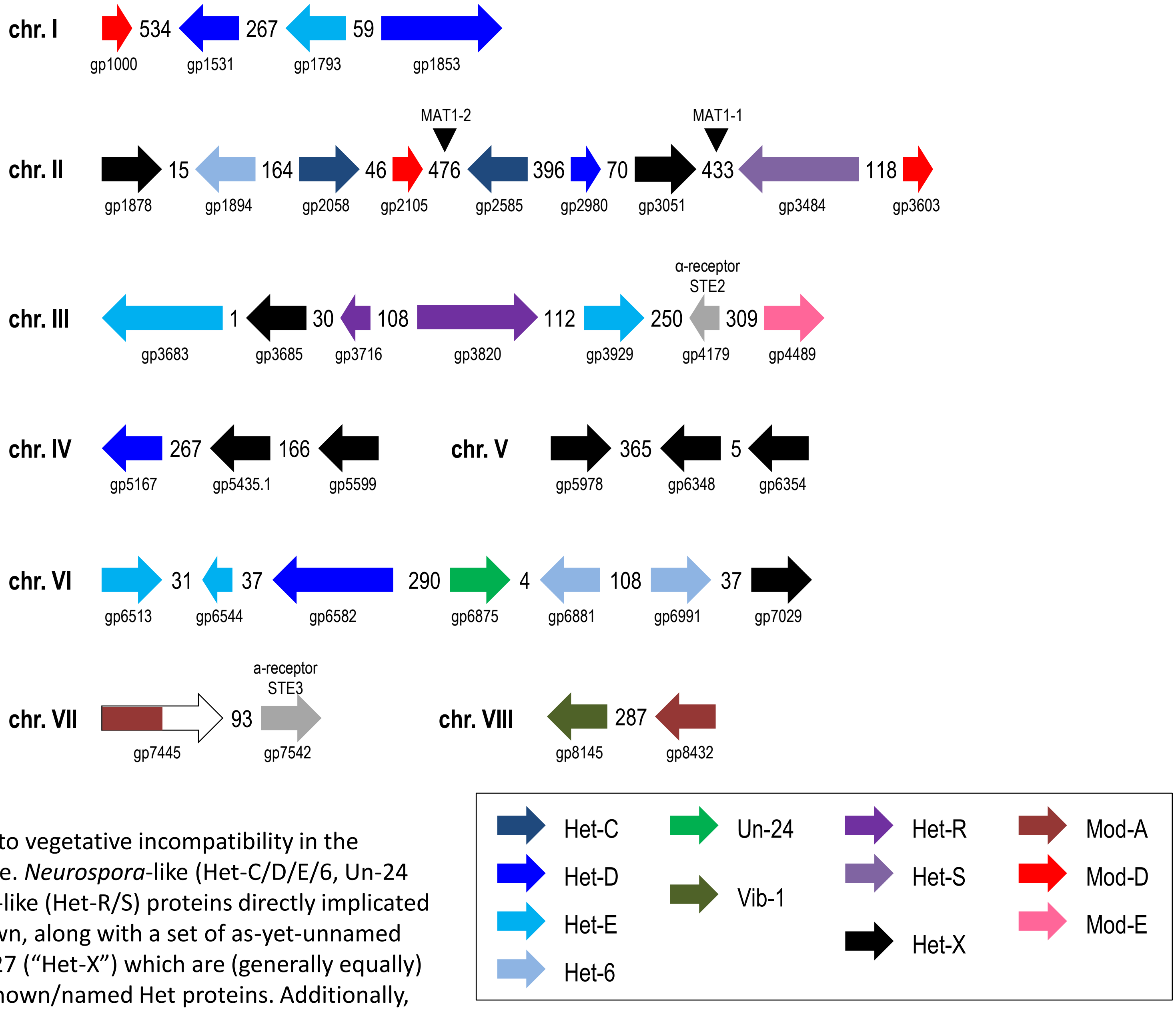

Fig. S7.—Proteins related to vegetative incompatibility in the *O. novo-ulmi* H327 genome. *Neurospora*-like (Het-C/D/E/6, Un-24 and Vib-1) and *Podospora*-like (Het-R/S) proteins directly implicated in incompatibility are shown, along with a set of as-yet-unnamed Het-like proteins from H327 ("Het-X") which are (generally equally) divergent from all other known/named Het proteins. Additionally, Mod ("modin") proteins (Mod-A/D/E) that are suppressors of vegetative incompatibility are shown. Finally, the locations of the mating-type genes on chromosome II are shown, along with the two mating-type pheromone receptors STE2 (α = type 1) and STE3 (a = type 2). For simplicity, sizes are roughly relative to final protein sizes (small = <500 aa, medium = 500-1000 aa, large = >1000 aa), not gene lengths, and the number of genes between proteins are enumerated.

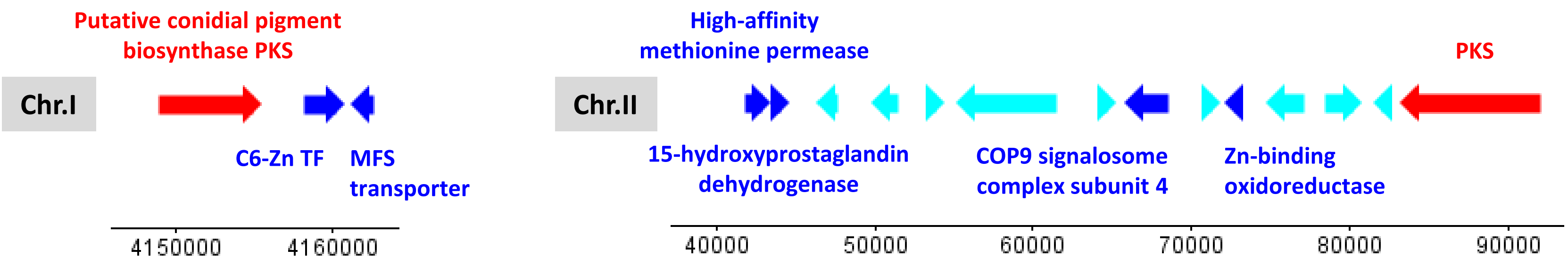

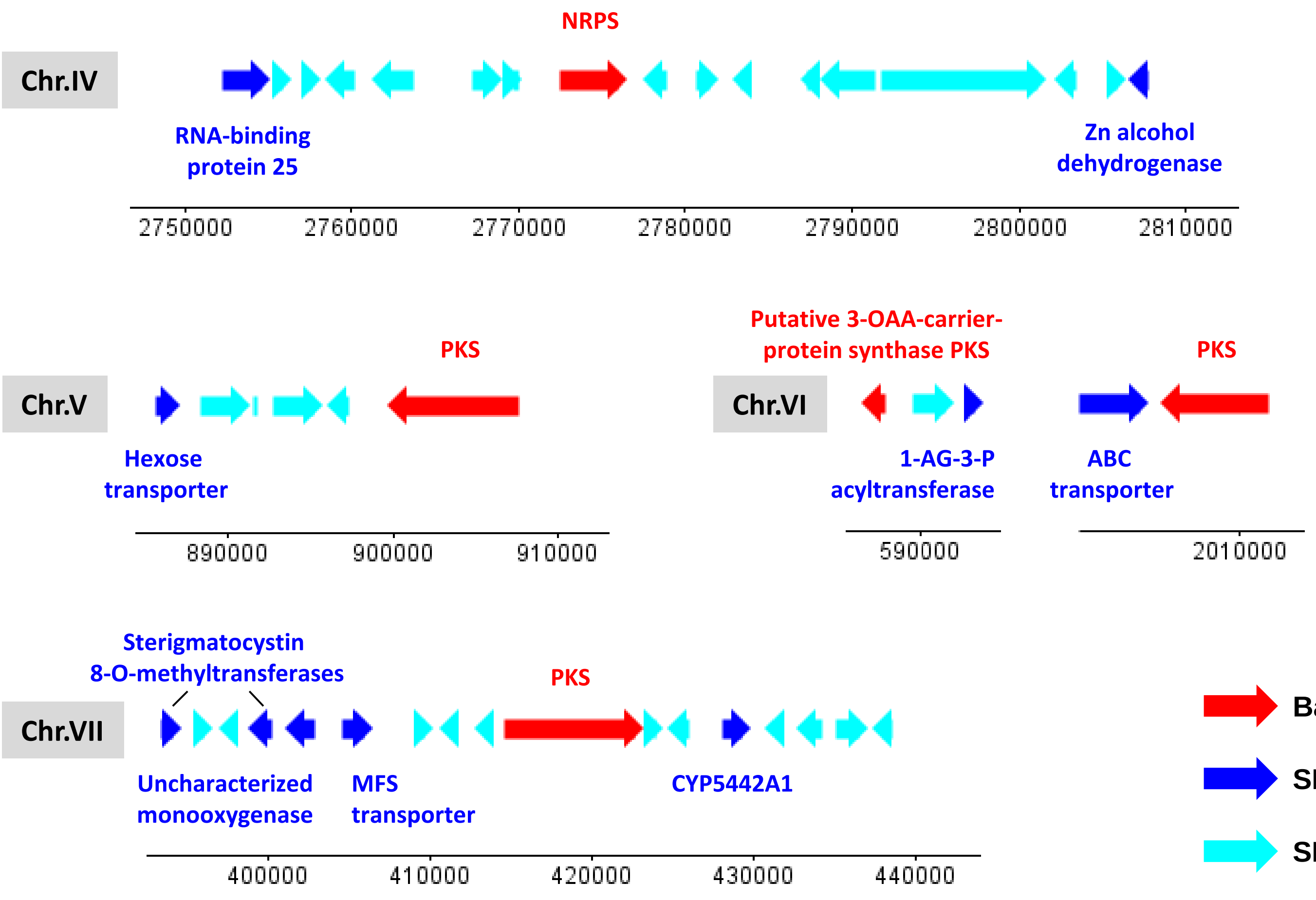

Fig. S8.—Locations and content of the secondary metabolite (SM) gene clusters in the *O. novo-ulmi* H327 genome. SM clusters were identified using the SMURF Program which finds “backbone” genes (PKS and NRPS in this case; red arrows/labels) around which to build clusters containing SM-domain-positive genes (likely involved in SM processes; blue arrows/labels). SM-domain-negative genes (cyan arrows) are peripheral and not likely to be functionally involved in the SM processes. Note that the scales show the chromosome coordinates of each cluster. ABC, ATP-binding cassette; 1-AG-3-P, 1-acylglycerol-3-phosphate; CYP, cytochrome P450; MFS, major facilitator superfamily; NRPS, non-ribosomal peptide synthase; 3-OAA, 3-oxoacyl-acyl; PKS, polyketide synthase; TF, transcription factor.

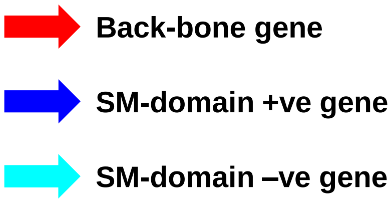

Supplement: Supplementary Data [file supp_evu281_suppl_data.zip › Comeau_etal-SupplFigs.pdf]
