# Supplementary material for: Functional Annotation of the Ophiostoma novo-ulmi Genome: Insights into the Phytopathogenicity of the Fungal Agent of Dutch Elm Disease
Source: Genome Biol Evol. 2014 Dec 24;7(2):410–30. doi: 10.1093/gbe/evu281 (PMC4350166; doi:10.1093/gbe/evu281)
Supplement: Supplementary Data [file supp_evu281_suppl_data.zip › Comeau_etal-SupplTablesS2-S6+S8.pdf]

Table S2

Primers Used for Re-Sequencing of *OPHIO3*, Novel Sequencing of MAT Loci and Cytochrome P450 (CYP) PCR/qPCR

Assay/use	Primer	Product size	Sequence (5'-3')
OPHIO3 amplification	OPHIO3chrIII-F	2002 bp	ATC TTG CAA CAC TTA ACT GTG TCT GG
(and sequencing)	OPHIO3chrIII-R		ACA AAC CGC GAA AGC TAA TCT CG
OPHIO3 sequencing	OPHIO3-NAN-Right	n/a	GGG CAG GGA AGA AAA ACA GAC A
	OPHIO3-NAN-RightRC	n/a	TGT CTG TTT TTC TTC CCT GCC C
MAT locus amplification	MATregionF	7700-9800 bp	AGT TAT TGA GAT CAA TAC CTC GGT TAA
(and sequencing)	MATregionR1		TCA GCA GAC ACG CAT TGT CTG
MAT locus sequencing	MAT111 orfsta1	n/a	ATG GCG CCA TCA ACG GCT
	MAT111 orfsta2A	n/a	ATG GTT AGT ATC CCT GTG G
	MAT111 orfsta3	n/a	ATG GAC CCA TGG CCT GA
	MAT111 orfsto1	n/a	TCA ACA AGG CCA ACC AAA G
	MAT111 orfsto2	n/a	TTG GAC GCC GAG CCA TAG T
	MAT111 orfsto3	n/a	TGC AAA GTT GAT AAA TGC AGT C
	MAT112 orfsta1A	n/a	ATG GAG CGC ACA GAA TCC
	MAT112 orfsta2	n/a	ATG GGC TTC GCC ATT GTA
	MAT112 orfsta3	n/a	ATG GAG GAT CGA CGA AC
	MAT112 orfsto1A	n/a	CTA GTC ATA GCG GCG GAT
	MAT112 orfsto2A	n/a	TCA CAA ACT GGA TAG GCT AG
	MAT113 3UTR	n/a	ACG GCT TTT GAA TTG GAG TAC TAT C
	MAT113 5UTR	n/a	TAC TTC ATC AAT CAG ATC CCA TC
	MAT113 ORF_Lg	n/a	ATG CCT ACC ACT ATC GAG
	MAT113 ORF_Rg	n/a	TCA GGA TCT AGC TGC TCC CAG TC
	MATreg400R	n/a	TTG CCA ATC AAC CAA GAT CTA G
	MATreg800R	n/a	CTG CTT TAT CCT CGG CAA C
	MATreg1400R	n/a	AAT GGC CGG ATA TGG ACT AG
	MATreg2000R	n/a	CAG CAG CTG TCA CAT GAG
	MATreg2600R	n/a	TGC AAC ACG GTC TGG CA
	MATreg4000R	n/a	GGA TTC TGT GCG CTC CAT
	MATreg4800F	n/a	ATC CGC CGC TAT GAC TAG
	MATreg5700R	n/a	AGC CGT TGA TGG CGC CAT
	MATreg6300F	n/a	CAA GGC ATA GGC CAA CAG

	MATreg6300F1	n/a	CAA AGC ATA GGT CAG CAG
	MATreg7500R	n/a	CTC AAG TGG CCA CTA TCA C
	MATreg7550R	n/a	GGA CGA GTG TTT GCA GGA G
	MATreg8000F	n/a	CTT TGG TTG GCC TTG TTG A
	MATreg8600F	n/a	GAA ATC CAC TGT AGC CCA CA
	MATreg8800R	n/a	TGC AGG TAC TAA GCA TGG ATT CTA
	MATreg9200F	n/a	AAA GAT ACA CTT CAG CGT AG
	MAT2reg3100F	n/a	CGG ATG CCG TCT CGA AAT C
	MAT2reg4600R	n/a	GTG TAT ATG AAG TTC AGA TCC AG
CYP *g2373* amplification	2373F	1010 bp	CGC AAG ACT ACC GTC TGC GTT GGT CCT A
	2373R		TGC GTT AAC CGA GTC CTT CTC CCA CC
CYP *g2373* qPCR	2373qF	280 bp	GGC CAT CCG TGA CCA GTT CGA CGA
	2373qR		GGC GAT CAT CAT GTG GGC AAT TTC GTG GTC
CYP *g7466* amplification	7466F	1000 bp	GGC AAG TCT ATC TCG CGC GAG TCT G
	7466R		CCG ATG GGA ACA TCT GGC TCG TCA
CYP *g7466* qPCR	7466qF	280 bp	GAT CAG GCA CTC AAC GTG CTG CTT GCT G
	7466qR		GCC GCC AAC AGG CAG GAC CGT A

Table S3

O. novo-ulmi H327 RNA-seq Summary and Comparison between Newbler and TopHat Mappers

	Newbler			TopHat		
Parameter	Yeast	Mycelial	Combined[a]	Yeast	Mycelial	Combined[a]
A. Illumina reads – raw[b]	15,334,986	12,406,022	27,741,008	15,334,986	12,406,022	27,741,008
B. Illumina reads – filtered[b]	6,807,900	6,313,935	13,121,835	6,807,900	6,313,935	13,121,835
C. Mapping to whole genome:						
- total reads mapped	5,836,596	5,463,131	11,299,727	6,368,442	5,986,484	12,354,926
- % reads mapped[c]	86%	87%	86%	94%	95%	94%
- mean map length	96 nt	97 nt	96 nt	96 nt	97 nt	96 nt
- mean coverage depth	18x	17x	34x	19x	18x	37x
D. Mapping to exons+introns:						
- total reads mapped	4,080,393	3,770,922	7,851,315	4,533,051	4,131,088	8,664,139
- % reads mapped[d]	70%	69%	69%	71%	69%	70%
- mean map length	96 nt	96 nt	96 nt	96 nt	96 nt	96 nt
- mean coverage depth	25x	24x	49x	28x	26x	54x
E. Mapping to exons only:						
- total reads mapped	4,132,580	3,867,910	8,000,490	4,430,603	4,067,485	8,498,088
- % reads mapped[d]	71%	71%	71%	70%	68%	69%
- mean map length	96 nt	97 nt	96 nt	96 nt	97 nt	96 nt
- mean coverage depth	28x	26x	53x	30x	27x	57x
F. Gene models with ≥1 mapped read (%)[e]	–	–	–	8417 (97%)	8432 (98%)	8521 (99%)
G. Gene models with ≥20 mapped read (%)[e]	–	–	–	7331 (85%)	7440 (86%)	7649 (89%)
H. Mean number of reads per gene[e]	–	–	–	491	459	475

[a]These values are alternatively the sums or the non-redundant totals of the yeast + mycelial samples, where appropriate.
[b]These values are the same for both Newbler and TopHat as they are the original input reads identical for both programs.
[c]Relative to the original filtered input reads above (B).
[d]Relative to only the reads that successfully mapped to the whole genome above (C).
[e]These parameters were only calculated/performed for TopHat as it showed superior total reads mapped (C-E).

Table S4

O. novo-ulmi H327 Chromosome Summary

Chr	Size (Mb)	#N (kb)[a]	%GC	%repeats[b]	tRNAs	rRNAs	CDS
I	6.938	21.2	50.1	2.37	51	13×5S	1,867
II	6.818	12.6	50.3	3.62	46	10×5S	1,825
III	3.670	9.7	50.1	3.39	28	2×5S	992
IV	3.420	8.3	50.0	2.41	34	2×5S	970
V	2.849	8.1	49.9	4.07	23	4×5S	801
VI	2.802	6.0	49.9	2.92	30	6×5S	760
VII	2.758	11.2	50.1	3.90	22	5S/18+5.8+28S	758
VIII	2.531	6.4	49.7	5.92	20	5×5S	667
TOTALS	31.785	83.6	50.1	3.38	254	46[c]	8,640

[a]The cumulative size of all gaps in the final assembly for each chromosome.

[b]Percentage of each chromosome sequence occupied by the repeats presented in Table 2.

[c]As the final size of the 18+5.8+28S tandem array has not been accurately determined, this number is a lower boundary.

Table S5

O. novo-ulmi H327 Changes to Gene Model Predictions After RNA-seq Correction

Parameter	Number	% of initial	% of final
Initial gene model set	8,525	100%	98.7%
Confirmed loss of genes:	53	0.6%	0.6%
- spurious genes to delete	36	0.4%	0.4%
- single genes fused to another[a]	17	0.2%	0.2%
Confirmed new genes:	168	2.0%	1.9%
- gene discovery	25	0.3%	0.3%
- “new” genes from splits[b]	143	1.7%	1.7%
Existing gene corrections:	756	8.9%	8.8%
- genes are longer from above fusions	17	0.2%	0.2%
- genes are longer[c]	135	1.6%	1.6%
- genes are shorter from above splits	134	1.6%	1.6%
- genes are shorter[c]	316	3.7%	3.7%
- internal changes only	154	1.8%	1.8%
Final validated gene set	8,640	101%	100%

[a]Fusions led to genes being “lost” to individual counts, but the coding regions are retained as part of the new fused genes.

[b]Similar to fusions, 2-4 “new” genes were created in number after each split (from 134 events below), but from (primarily) existing coding regions.

[c]Can also include additional internal changes as well.

Table S6

OPHIO Elements in the *O. novo-ulmi* H327 Genome (see Figure 4) and the Effect of RIP (Repeat-Induced Point Mutation) on Dinucleotide Changes

						Mutations relative to model (% dinucleotides mutated)			
Chr	Element	Topology	State[a]	Size (bp)[b]	%AT	CpA↔TpA + TpG↔TpA	CpC↔TpC + GpG↔GpA	CpG↔TpG + CpG↔CpA	CpT↔TpT + ApG↔ApA
Relative to the originally-sequenced O. novo-ulmi OPHIO1 *(DQ649003.1) as model*									
model	*OPHIO1*	stand-alone	functional	1865	56.9	–	–	–	–
III	*OPHIO1*	stand-alone	functional	1865	56.9	4 (2%)	0 (–)	4 (4%)	0 (–)
V	*OPHIO1*	split	degraded	1863	65.4	88 (39%)	2 (1%)	5 (5%)	45 (18%)
Relative to the originally-sequenced O. ulmi OPHIO2 *(DQ649004.1) as model*									
model	*OPHIO2*	stand-alone	functional	1865	57.2	–	–	–	–
VII	*OPHIO2*	split	degraded	1867	65.7	91 (43%)	4 (3%)	7 (7%)	55 (22%)
Relative to the originally-sequenced O. novo-ulmi OPHIO3 *(DQ649005.1) as model*									
model	*OPHIO3*	stand-alone	degraded	1849	58.4	–	–	–	–
III	*OPHIO3*	stand-alone	degraded	1850	58.4	43 (26%)	1 (<1%)	4 (4%)	25 (9%)
VII	*OPHIO3*	split	degraded	1850	66.4	91 (55%)	4 (3%)	6 (6%)	84 (30%)
VIII	*OPHIO3*	split	degraded	1841	73.2	96 (59%)	4 (3%)	6 (6%)	134 (48%)
VIII	*OPHIO3*	stand-alone	degraded	1896	71.7	81 (49%)	9 (6%)	15 (15%)	105 (37%)

[a]*In silico* assessment of whether the elements are still functional or degraded to the point of not functioning (accumulated stop codons in their transposases).

[b]For the elements split in two by intervening DNA, this size represents the reconstructed size when left- and right-halves were recombined.

Note.—All dinucleotide changes were calculated using RIPCAL (Hane and Oliver 2008).

Hane JK, Oliver RP. 2008. RIPCAL: A tool for alignment-based analysis of repeat-induced point mutations in fungal genomic sequences. BMC Bioinformatics. 9:478.

Table S8

Cytochrome P450s (CYP) in *O. novo-ulmi* H327 (Unique Families in Blue) Compared to *Grosmannia clavigera* kw1407 (Unique Families in Red)

CYP family	Putative activity/pathway	*O. novo-ulmi* members	*G. clavigera* members
CYP51	Universal - membrane ergosterols	CYP51F1	CYP51F1
**CYP52**	*Pinene/limonene DEG/UTIL?*	**CYP52P6**	–
CYP53	Benzoate/phenolics DEG	CYP53E2	CYP53A27, CYP53E1
**CYP54**	Ent-kaurene SYN	**CYP54C7**	–
CYP61	Universal - membrane ergosterols	CYP61A1	CYP61A1
CYP65	Benzoate/phenolics/pisatin DEG<br>Trichothecene/averantin SYN	CYP65BB4, CYP65CD1, CYP65CE1, CYP65CF1	CYP65BJ1, CYP65BJ2, CYP65BJ3
CYP504	Phenylacetates DEG	CYP504E13	CYP504A22, CYP504E10
CYP505	Fatty acid hydroxylation	CYP505A28, CYP505S1	CYP505A22
**CYP526**	?	–	**CYP526B2**
**CYP527**	Pisatin DEG	**CYP527K1**	–
CYP528	Benzoate/phenolics/pisatin DEG	CYP528C2	CYP528C1
CYP529	*Terpene DEG? (GcRNAseq)*	CYP529A5	CYP529A3
**CYP530**	(Methyl)sterigmatocystin SYN<br>*Terpene DEG? (GcRNAseq)*	–	**CYP530A13**
**CYP531**	Benzoate/phenolics/pisatin DEG<br>*Terpene DEG? (GcRNAseq)*	–	**CYP531A6**
CYP532	Pisatin DEG<br>*Triglyceride/oleic acid UTIL? (GcRNAseq)*	CYP532A14, CYP532H6	CYP532A10
CYP533	(Methyl)sterigmatocystin SYN	CYP533C2	CYP533C1
CYP534	?	CYP534F2	CYP534F1
**CYP536**	Pisatin DEG	**CYP536A6**	–
CYP537	Benzoate/phenolics DEG	CYP537A11, CYP537D4, CYP537E2	CYP537A8, CYP537D3, CYP537E1
CYP539	Alkane DEG<br>*Triglyceride/oleic acid UTIL? (GcRNAseq)*	CYP539A17	CYP539A11
**CYP540**	?	–	**CYP540A6, CYP540B22**
CYP544	?	CYP544A9	CYP544A7
CYP546	?	CYP546A4	CYP546A3

CYP548	Trichothecene SYN	CYP548A17	CYP548A12
**CYP552**	Trichothecene SYN<br>*Terpene DEG? (GcRNAseq)*	–	**CYP552A3**
CYP559	?	CYP559B3	CYP559B2
CYP570	Benzoate/phenolics/pisatin DEG	CYP570A4, CYP570B4	CYP570A3, CYP570B2
**CYP574**	?	–	**CYP574B1**
CYP577	Benzoate/phenolics DEG	CYP577A16	CYP577A12
CYP578	Pisatin DEG	CYP578M1	CYP578E2
CYP582	?	CYP582D1	CYP582C1
**CYP587**	?	–	**CYP587D1**
**CYP590**	?	**CYP590B1**	–
CYP602	Ent-kaurene SYN	CYP602B7	CYP602B4
**CYP609**	?	**CYP609D1**	–
CYP617	?	CYP617A9	CYP617A6, CYP617D10
**CYP620**	(Methyl)sterigmatocystin SYN	**CYP620T1**	–
CYP621	(Methyl)sterigmatocystin SYN	CYP621D3	CYP621C2, CYP621D1
**CYP624**	Benzoate/phenolics DEG	–	**CYP624B1**
**CYP628**	Benzoate/phenolics/pisatin DEG<br>Averantin SYN<br>*Terpene DEG? (GcRNAseq)*	–	**CYP628B2, CYP628K1**
**CYP630**	Benzoate/phenolics DEG<br>*Triglyceride/oleic acid UTIL? (GcRNAseq)*	–	**CYP630B18**
**CYP637**	*Triglyceride/oleic acid UTIL? (GcRNAseq)*	–	**CYP637C1**
**CYP654**	?	–	**CYP654C1**
**CYP673**	Benzoate/phenolics DEG	**CYP673C5**	–
**CYP5047**	(Methyl)sterigmatocystin SYN	–	**CYP5047B1**
**CYP5054**	Phenylacetates DEG	–	**CYP5054A3**
CYP5062	Benzoate/phenolics DEG	CYP5062A4	CYP5062A3
**CYP5077**	Pisatin DEG	**CYP5077C1**	–
CYP5128	Benzoate/phenolics DEG	CYP5128E2	CYP5128E1
**CYP5269**	*Terpene DEG? (GcRNAseq)*	–	**CYP5269A3**
**CYP5282**	?	–	**CYP5282D1**
**CYP5338**	?	–	**CYP5338A1**
**CYP5386**	?	**CYP5386B1**	–

**CYP5442**	(Methyl)sterigmatocystin SYN?	**CYP5442A1**	–
**CYP6001**	Linoleate diol SYN	**CYP6001C18**	–
**CYP6006**	?	**CYP6006A1**	–

Note.—*Grosmannia* CYP450s and RNA-seq data were compiled from the analysis of Lah et al (2013). DEG, degradation; GcRNAseq, *G. clavigera* RNA-seq evidence; SYN synthesis; UTIL, utilization.

Lah L, Haridas S, Bohlmann J, Breuil C. 2013. The cytochromes P450 of *Grosmannia clavigera*: Genome organization, phylogeny, and expression in response to pine host chemicals. Fungal Genet Biol. 50:72-81.
